# Phenotypic and functional characterisation of the luminal cell hierarchy of the mammary gland

**DOI:** 10.1186/bcr3334

**Published:** 2012-10-22

**Authors:** Mona Shehata, Andrew Teschendorff, Gemma Sharp, Nikola Novcic, I Alasdair Russell, Stefanie Avril, Michael Prater, Peter Eirew, Carlos Caldas, Christine J Watson, John Stingl

**Affiliations:** 1 Mammary Stem Cell Laboratory, Cancer Research UK, Cambridge Research Institute, Li Ka-Shing Centre, Robinson Way, Cambridge CB2 0RE, UK; 2 Statistical Cancer Genomics, UCL Cancer Institute, University College London, 72 Huntley Street, London WC1E 6BT, UK; 3 Present address: Department of Pathology, Technical University Munich, 21 Arcis Street, Munich 80333, Germany; 4 Department of Molecular Oncology, British Columbia Cancer Research Centre, 675 West 10th Avenue, Vancouver V5Z 1L3, Canada; 5 Department of Oncology, University of Cambridge, Worts Causeway, Cambridge CB1 9RN, UK; 6 Cambridge Breast Unit, Addenbrooke's Hospital, Cambridge University Hospital NHS, Foundation Trust and NIHR Cambridge Biomedical Research Centre, Box 97 Hills Road, Cambridge CB2 2QQ, UK; 7 Cambridge Experimental Cancer Medicine Centre, Cancer Research UK, Cambridge Research Institute, Li Ka-Shing Centre, Robinson Way, Cambridge CB2 0RE, UK; 8 Breast Cancer Functional Genomics Laboratory, Cancer Research UK, Cambridge Research Institute, Li Ka-Shing Centre, Robinson Way, Cambridge CB2 0RE, UK; 9 Department of Pathology, University of Cambridge, Tennis Court Road, Cambridge CB2 1QP, UK

## Abstract

**Introduction:**

The organisation of the mammary epithelial hierarchy is poorly understood. Our hypothesis is that the luminal cell compartment is more complex than initially described, and that an understanding of the developmental relationships within this lineage will help in understanding the cellular context in which breast tumours occur.

**Methods:**

We used fluorescence-activated cell sorting along with *in vitro *and *in vivo *functional assays to examine the growth and differentiation properties of distinct subsets of human and mouse mammary epithelial cells. We also examined how loss of steroid hormones influenced these populations *in vivo*. Gene expression profiles were also obtained for all the purified cell populations and correlated to those obtained from breast tumours.

**Results:**

The luminal cell compartment of the mouse mammary gland can be resolved into nonclonogenic oestrogen receptor-positive (ER^+^) luminal cells, ER^+ ^luminal progenitors and oestrogen receptor-negative (ER^-^) luminal progenitors. The ER^+ ^luminal progenitors are unique in regard to cell survival, as they are relatively insensitive to loss of oestrogen and progesterone when compared with the other types of mammary epithelial cells. Analysis of normal human breast tissue reveals a similar hierarchical organisation composed of nonclonogenic luminal cells, and relatively differentiated (EpCAM^+^CD49f^+^ALDH^-^) and undifferentiated (EpCAM^+^CD49f^+^ALDH^+^) luminal progenitors. In addition, approximately one-quarter of human breast samples examined contained an additional population that had a distinct luminal progenitor phenotype, characterised by low expression of ERBB3 and low proliferative potential. Parent-progeny relationship experiments demonstrated that all luminal progenitor populations in both species are highly plastic and, at low frequencies, can generate progeny representing all mammary cell types. The ER^- ^luminal progenitors in the mouse and the ALDH^+ ^luminal progenitors in the human appear to be analogous populations since they both have gene signatures that are associated with alveolar differentiation and resemble those obtained from basal-like breast tumours.

**Conclusion:**

The luminal cell compartment in the mammary epithelium is more heterogeneous than initially perceived since progenitors of varying levels of luminal cell differentiation and proliferative capacities can be identified. An understanding of these cells will be essential for understanding the origins and the cellular context of human breast tumours.

## Introduction

Human breast tumours are very heterogeneous, with approximately five molecular subtypes recognised; these molecular subtypes are categorised as Luminal A, Luminal B, claudin^low^, basal-like and Her2 [1-3]. Currently unknown is whether breast tumours have a common cell of origin or whether different types of tumours originate from different types of cells, or whether it is a combination of these two processes [4]. Support for the second hypothesis comes from studies in which different populations of human breast epithelial cells were selected from *in vitro *cultures or were purified using flow cytometry and reverse-engineered into tumours of distinct subtypes using lentiviral vectors [5,6]. Similar results have been observed in a mouse model where loss of Brca1 in different epithelial cell compartments resulted in tumours with different histologies [7]. An understanding of the properties of the normal mammary epithelial cell hierarchy will thus be important in understanding the cellular context in which human breast tumours occur. Similarly, an understanding of this hierarchy may also give insight into the properties of cancer stem cells and the behaviour of tumours during the emergence of therapeutic resistance.

The mammary epithelium has traditionally been described as a bilineage epithelium composed of luminal cells and basally-positioned myoepithelial cells that are collectively organised in a series of ducts that drain lobuloalveolar structures during lactation. Previous studies have demonstrated that mammary stem cells have features characteristic of basal cells, whereas the bulk of the progenitor cells display predominantly luminal features and have luminal-restricted development potential [8,9], although a recent report has demonstrated that a separate stem/progenitor cell maintains each lineage during adult tissue homeostasis [10]. The luminal cell compartment is heterogeneous since only a subset of these cells expresses oestrogen receptor (ER) [11]. Most of these ER^+ ^cells are perceived to be relatively mature cells since they are rarely observed to be cycling in adult mammary tissue [12,13]. However, rare proliferating ER^+ ^cells can be detected in the mouse mammary gland, suggesting the existence of an ER^+ ^progenitor cell [14]. More recently, fluorescence-activated cell sorting has been used to prospectively isolate luminal progenitors (LPs) from the mouse mammary gland based on differential expression of c-Kit and CD14 or c-Kit and Sca1 [15,16]. This latter study was able to identify a subpopulation of cells with a Sca1^+^c-Kit^+ ^phenotype that was enriched for ER^+ ^LP cells [15]. Similarly, an ER^- ^LP cell that has a CD24^high^Sca1^-^c-Kit^+ ^phenotype can also be identified [15,17]. These cells appear to function as alveolar progenitor cells and are characterised by high expression of the transcription factor Elf5 [18].

To further characterise the cells that make up the luminal cell hierarchy in both the human and mouse mammary glands, we used a combination of fluorescence-activated cell sorting, *in vitro *and *in vivo *functional assays and gene expression profiling strategies. Our results describe the prospective isolation and functional characterisation of discrete ER^+ ^and ER^- ^LP populations that are present in both species. Our results also demonstrate that both of these populations are developmentally plastic and display multilineage potential, and that the ER^+ ^LPs, at least in the mouse, have a relative survival advantage in a low oestrogen/progesterone environment. We also identify a novel breast cell type in the human mammary epithelium that is characterised by low expression of ERBB3.

## Materials and methods

### Dissociation of human and mouse mammary tissue

All primary human material was derived from 11 reduction mammoplasties at Addenbrooke's Hospital, Cambridge, UK, under full informed consent and in accordance with the National Research Ethics Service, Cambridgeshire 2 Research Ethics Committee approval (08/H0308/178) as part of the Adult Breast Stem Cell Study. All tissue donors had no previous history of cancer and were premenopausal (ages 18 to 46). Mammary tissue was dissociated to single cell suspensions as previously described [19].

The number 3 and/or number 4 mammary glands were dissected from 10-week-old to 14-week-old virgin or 20-day pregnant female C57BL6/J, C57BL6/J.CBA-Tg (ACTbEGFP) and FVB mice and were dissociated in DMEM/F12 (with 2.5 mM L-glutamine and 15 mM HEPES; Gibco, Paisley, Renfrewshire, UK) supplemented with 1 mg/ml collagenase (Roche, Burgess Hill, West Sussex, UK), 100 U/ml hyaluronidase (Sigma, Poole, Dorset, UK) and 50 μg/ml gentamicin (Gibco) for 14 to 16 hours at 37°C. The mammary glands were then processed to single cells as previously described [8]. In some experiments, 8-week-old C57BL/6J mice were ovariectomised or sham-operated 3 weeks prior to collection of mammary tissue.

### Flow cytometry

Single cell suspensions of human mammary cells were treated to detect the enzyme activity of aldehyde dehydrogenase (ALDH) using the Aldefluor Kit (StemCell Technologies, Grenoble, Rhône-Alpes, France) as per the manufacturer's instructions. The cells were then preblocked with 10% normal rat serum (Sigma) and incubated with the following primary antibodies (Table S1 in Additional file 1): CD31-PE/Cy7, CD45-PE/Cy7, epithelial cell adhesion molecule (EpCAM)-PE, CD49f-Alexa Fluor (AF) 647 or CD49f-Pacific Blue, ERBB3-biotin, CD44-AF647, MUC1-AF647, and CD24-AF647. Hank's balanced salt solution supplemented with 2% FBS (Gibco) was used as the diluent for all preblock, antibody incubation and washing steps.

Mouse mammary cells were preblocked with 10% normal rat serum and then incubated with the following primary antibodies (Table S1 in Additional file 1): CD31-biotin, CD45-biotin, Ter119-biotin, BP-1-biotin, EpCAM-AF647, CD49f-AF488, or CD49f-Pacific Blue, CD49b-PE and Sca1-PE/Cy7. CD45, Ter119, CD31 and BP-1 were used to deplete contaminating haematopoietic cells, endothelial cells and a proportion of stromal cells, respectively (collectively termed Lin^+ ^cells). Where required, single cell suspensions of mouse mammary cells were treated to detect the enzyme activity of ALDH using the Aldefluor Kit as per the manufacturer's instructions, and then cells were stained as above.

Biotin-conjugated antibodies were detected with streptavidin-APC-Cy7 (BioLegend, Bar Hill, Cambridgeshire, UK). Cells were then filtered through a 30 μm cell strainer and incubated with 4',6-diamidino-2-phenylindole (Invitrogen, Paisley, Renfrewshire, UK) or propidium iodide (Sigma). Human cells were sorted using an Influx (Becton Dickinson, Oxford, Oxfordshire, UK) and mouse cells were analysed using an LSRII (Becton Dickinson) and they were sorted on a FACSAria I (Becton Dickinson) or a MoFlo (Beckman Coulter, High Wycombe, Buckinghamshire, UK). The gating cascade is shown in Additional file 2. A cell recovery count was performed after each sort. Single-stained control cells were used to perform compensation manually. Gates were set in reference to negative controls stained with isotype antibodies conjugated to individual fluorochromes or to fluorescence-minus-one controls (omitting one reagent at a time). The ALDH^+ ^gate was set in reference to control populations incubated with the ALDH inhibitor DEAB in addition to Aldefluor. Flow cytometry data were analysed using FlowJo™ software (Tree Star, Inc., Ashland, OR, USA).

### Renal grafting and mouse mammary repopulating unit assays

All animal work was approved by Cambridge Research Institute Animal Ethics Committee and the Home Office. Renal capsule experiments were carried out on 10-week-old female NOD/SCID IL2Rγc^-/- ^(NSG) mice as previously described [19] with the modification that in some experiments the collagen gels were supplemented with 20% growth factor-reduced Matrigel (BD Biosciences, Oxford, Oxfordshire, UK). A sialastic pellet containing 2 mg 17β-oestradiol and 4 mg progesterone was implanted subcutaneously in recipient mice when human cells were being transplanted [20]. In some experiments, the hormone pellets were surgically excised 5 weeks post surgery and the mice mated. To recover renal gels, recipient mice were killed and the retrieved gels were fixed in 4% paraformaldehyde for 1 hour before being processed into paraffin. On occasion, gels were dissociated for 4 to 5 hours at 37°C in Mouse EpiCult-B™ media (StemCell Technologies) supplemented with 5% FBS, 600 U/ml collagenase and 200 U/ml hyaluronidase. After digestion, cells were washed in Hank's balanced salt solution supplemented with 2% FBS, trypsinised for 5 minutes with gentle pipetting and injected into the cleared mammary fat pads of NSG mice as described below.

For the mouse mammary repopulating unit (MRU) assays, donor cells were suspended in 65% Hank's balanced salt solution supplemented with 2% FBS additionally supplemented with 25% growth factor-reduced Matrigel and 10% trypan blue solution (0.4%; Sigma), such that a 10 μl injection volume contained the desired cell dose. The endogenous mammary epithelium in the inguinal glands of 3-week-old female C57BL6/J or NSG mice was cleared and cells were injected into cleared fat pads as previously described [21]. The mice were mated 3 weeks after surgery and the number 4 glands were removed during pregnancy and fixed in Carnoys fixative and stained with carmine alum. An outgrowth was scored positive if it contained both lobular and ductal elements. MRU frequencies were calculated using the Extreme Limiting Dilution Analysis tool [22]). In some experiments, mice were kept in a virgin state for a total of 10 weeks and the number 4 glands removed for analysis by flow cytometry or histology.

### *In vitro *colony-forming assays

Flow-sorted human mammary cells were seeded into 60 mm plates with 2.5 × 10^5 ^irradiated NIH-3T3 feeder cells. The cultures were maintained in Human EpiCult-B (StemCell Technologies) supplemented with 5% FBS (StemCell Technologies) and 50 μg/ml gentamicin for 24 to 48 hours and then the media changed to serum-free conditions and maintained for an additional 10 to 12 days. Flow-sorted mouse cells were cultured in Mouse EpiCult-B and 50 μg/ml gentamicin in the presence of irradiated feeders for 5 to 7 days. At the end of the assays, the colonies were fixed with acetone:methanol (1:1), stained with Giemsa (Fisher Scientific, Cramlington, Northumberland, UK) and enumerated under a microscope.

In some experiments, the sorted cells were seeded within growth factor-reduced Matrigel and cultured in the presence of Human or Mouse EpiCult-B and irradiated feeders for 14 to 21 days. In some experiments, the culture media were changed after 7 days into differentiation media (DMEM/F12 with Glutamax (Gibco) supplemented with 10% FCS, 1 μM dexamethasone, 5 μg/ml insulin and 5 μg/ml prolactin) to induce lactogenic differentiation of the mammary cells, and the cultures were maintained for a further 7 to 14 days. At the end of the assay, the gels were then fixed in 4% paraformaldehyde and embedded in paraffin for sectioning and immunostaining.

### Immunofluorescence and immunohistochemistry

Sorted cells were allowed to adhere to μ-Slide eight-well chamber poly-lysine-coated slides (Ibidi, Uddingston Glasgow, UK) for 15 minutes before fixation in 4% paraformaldehyde. Cells were blocked in 10% normal goat serum (Sigma) for 1 hour and stained with primary antibodies (Table S1 in Additional file 1) overnight at 4°C. Goat anti-mouse or anti-rabbit antibody conjugated to either AF488 or AF555 (Invitrogen) was used to detect primary antibodies. IgG antibodies at the same concentration as the primaries were used as isotype controls. Slides were stained with 4',6-diamidino-2-phenylindole to visualise the nuclei. Paraffin-embedded renal gels and Matrigel cultures were sectioned at 4 μm, deparaffinised and boiled in pH 6.0 citrate buffer. The sections were stained as above. Where required, a Mouse on Mouse (Vector Labs, Peterborough, Cambridgeshire, UK) preblocking kit was used as per the manufacturer's instructions.

### RNA preparation, quantitative RT-PCR analysis

Freshly sorted cells were pelleted and the supernatant removed. RNA was extracted using the PicoPure™ RNA extraction kit (Applied Biosystems, Paisley, Renfrewshire, UK) as per the manufacturer's instructions, and samples were treated with DNase using the RNase-free DNase Set (Qiagen, Crawley, West Sussex, UK). For mouse quantitative RT-PCR analysis, RNA from sorted cells of five independent experiments was collected. For human quantitative RT-PCR analysis, RNA from sorted cells of six different human breast specimens was collected. cDNA was generated using 100 ng RNA and random hexamers in a 20 μl reaction using SuperScript III (Invitrogen) according to the manufacturer's instructions. cDNA was diluted 1/10 and 1 μl was used in a 10 μl volume reaction with 2× SYBR Green Fast PCR Master Mix (Applied Biosciences) and 1 μl of 5 μM forward and reverse primers (Table S2 in Additional file 1) and H_2_O. The real-time PCR reactions for each sample were performed in triplicate with an ABI 7900 Real Time PCR system under the following conditions: 95°C for 20 seconds followed by 40 cycles of 95°C for 1 second and 60°C for 20 seconds, followed by a dissociation run to obtain melt profiles of the amplicons. A no-template control (no cDNA) was used as a control for all primers, also performed in triplicate. Results were analysed with the delta-delta method normalised to two housekeeping genes (Ppia and Rpl13a or UBC and TBP for mouse and human samples, respectively) and compared with a comparator sample (nonclonogenic luminal (NCL) cells).

### Microarrays

Total RNA was purified from freshly sorted cell populations using the PicoPure™ RNA extraction kit. Up to 250 ng RNA was labelled according to the standard Illumina gene expression array protocols with the Ambion TotalPrep 96 kit (4397949; Ambion, Paisley, Renfrewshire, UK). Biotinylated complementary RNA was quality controlled using Agilent Bioanalyser and quantified by spectrophotometry (Nanodrop, Ringmer, East Sussex, UK), and 750 ng cRNA was hybridised to Illumina Mouse6 or HumanHT12v4 BeadChips and washed, stained and scanned according to the standard protocol (WGGX DirectHyb Assay Guide 11286331 RevA; Illumina, Saffron Walden, Essex, UK). Arrays were scanned on an Illumina BeadArray scanner, and data were processed using the Bioconductor beadarray package [23]. (Further information can be found in Additional file 3.) All data files can be accessed via the Gene Expression Omnibus [GEO:GSE35399].

### Correlation of normal cell subpopulations with breast cancer datasets

#### Centroid construction

Centroids of gene expression for each cell subpopulation were built from the union set of top differentially expressed genes between each pair of cell subtypes. To identify differentially expressed genes we first filtered genes according to variability and then used the limma R package to rank them according to differential expression using *B *statistics. The False Discovery Rate was estimated using the *q*-value R package. To avoid skewing the number of centroid genes to specific cell types, we selected the top 250 upregulated and top 250 downregulated genes in each cell-type comparison. All of these passed False Discovery Rate corrected *P *< 0.05. Since the three LP subpopulations were more similar to each other than to any of the other cell types, the corresponding centroids were constructed by the union set of the top 100 upregulated and downregulated genes for each of the three pairwise comparisons. A centroid for the whole LP population (including the three LP subpopulations) was also constructed. Finally, for each cell type the centroid was constructed by averaging the expression of each gene across the samples belonging to that cell type.

#### Correlation scores

Having constructed the cell-type-specific centroids, we next assessed their correlations to breast cancer profiles. First, each breast tumour profile was classified into one of the five intrinsic subtypes using the SSP predictor of Hu and colleagues [24] or the claudin^low ^subtype assigned by Herschkowitz and colleagues [25]. Second, each tumour gene expression profile was correlated to each of the centroids using a linear regression. Centroid profiles as well as tumour profiles were scaled to unit variance in order to ensure that regression coefficients, which reflect Pearson correlations, are comparable. Since each tumour can be thought of as a potential mixture of transformed cells from the different normal cell subpopulations, we also modelled each tumour profile as an explicit mixture of the normal cell centroids using a multivariate regression framework. In this framework, each regression coefficient represents a partial correlation and reflects the strength of association between the tumour profile and a given cell-type centroid after taking into account the contributions from the other cell types.

#### Decision tree classifier

We used a nearest centroid decision tree classifier to assign to each breast cancer a cell-type subpopulation according to how similar their tumour profile is to each of the cell centroids. To achieve this assignment we used a decision tree. First, each tumour was assigned to either the stromal, basal, luminal or LP centroid using the nearest centroid rule on the Pearson correlation scores. If a sample classified according to the LP type, we then assigned it to a further LP subtype using the nearest centroid rule against the correlation scores computed from the individual LP subtype-specific centroids.

### Statistical analysis

Data presented are the mean of multiple independent experiments and the standard error of the mean. One-way analysis of variance was used to test multiple groups followed by Tukey's post test to test significant differences between pairs of results. Comparisons between just two groups were analysed by Student's *t *test. Significance was set at * = *P *< 0.05, ** = *P *< 0.01 or *** = *P *< 0.0001.

## Results

### Two distinct luminal progenitor cell types exist within the mouse mammary epithelium

**Figure 1:**
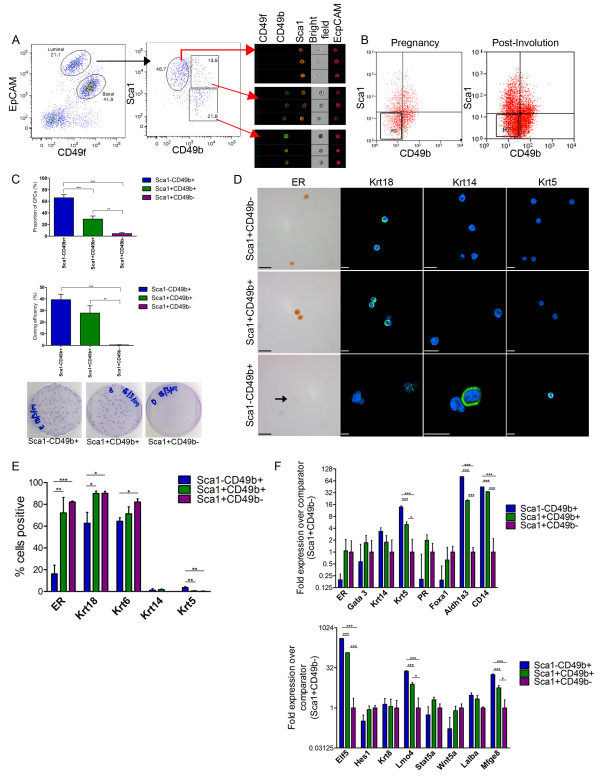
**Mouse luminal compartment contains distinct subpopulations**. **(A) **Distribution of epithelial cell adhesion molecule (EpCAM) and CD49f among Lin^- ^cells resolves the luminal and basal cell subpopulations (left). Expression of Sca1 and CD49b among the luminal cells resolves three subpopulations (middle), whose morphology can be visualised using the Image Stream™ analyser (Merck Millipore, Watford, Hertfordshire, UK) (right). **(B) **Expression of Sca1 and CD49b in pregnant (left; R9 gate) and 6 weeks post involution (right; R7 gate) mammary glands indicating the emergence of a fourth luminal cell population. **(C) **Bar chart showing the distribution of colony-forming cells (CFCs; top) and cloning efficiencies (middle) of the three luminal populations. Images of CFCs indicating only CD49b^+ ^cells can form colonies (bottom). **(D) **Immunocytochemical and immunofluorescence analysis of mouse luminal subpopulations. Cells were sorted, fixed onto slides and stained to detect oestrogen receptor (ER), keratin (Krt)18, Krt14 and Krt5. **(E) **Bar chart showing the percentage positive cells for each population. **(F) **Gene expression analysis of Sca1^-^CD49b^+ ^and Sca1^+^CD49b^+ ^populations relative to the comparator (Sca1^+^CD49b^-^) for luminal and basal transcripts. Error bars for all indicate the standard error of the mean for six independent experiments. *P < 0.05, **P < 0.01, ***P < 0.0001. Scale bars = 10 μm.

To test the hypothesis that the LP population is a heterogeneous population, we dissociated mammary glands from 10-week-old virgin C57BL6/J females and analysed the liberated cells using flow cytometry to detect EpCAM, and CD49f (α_6_-integrin; Figure 1A and Additional file 2A). We used EpCAM rather than the previously described CD24 [8,9] since the use of EpCAM permits greater resolution of the luminal and basal cell subpopulations. We also used CD49b (α_2_-integrin) instead of CD61 (β_3_-integrin) to identify LPs. We observed that CD49b^+ ^was a more selective marker of LPs than the previously reported CD61 [26] since up to 47% of progenitors are of CD61^- ^phenotype (Additional file 4A,B).

As shown in Figure 1A, the luminal cell compartment can be subdivided into three distinct subpopulations on the basis of expression of CD49b and Sca1. We observed a fourth population (Sca1^-^CD49b^-^) during pregnancy and remnants of this population are maintained throughout the involution and post-involution stages (region gates R9 and R7 in Figure 1B; see Additional file 4C). Flow sorting the three subpopulations from the virgin gland and seeding them into two-dimensional colony-forming cell (CFC) assays reveals that progenitor activity is restricted to the Sca1^-^CD49b^+ ^and Sca1^+^CD49b^+ ^subpopulations (Figure 1C). Cloning efficiency was observed to be approximately 25% and 40% for Sca1^+^CD49b^+ ^and Sca1^-^CD49b^+ ^progenitors, respectively. However, we suspect that these cell populations may be pure progenitor cells, since flow-associated toxicity is calculated to be as high as 75% (Additional file 4D).

Previously, gene expression profiling of sorted mouse mammary epithelial cells identified CD14, a co-receptor for bacterial lipopolysaccharide, as being highly enriched in the luminal cell population [8]. When we examined the distribution of CD14 expression among the luminal population, we observe that approximately 86% of LP cells express this protein, whereas CD14^- ^luminal cells are relatively deficient in CFCs (Additional file 4E,F,G). Other studies have reported that c-Kit expression identifies both ER^- ^and ER^+ ^LPs [15] and progenitors that are primed to generate progeny that can synthesise milk proteins [16]. When examining c-Kit expression in luminal cells, we observed variation between mouse strains, with c-Kit expression localised to a minority of Sca1^+ ^luminal cells, and only in FVB mice (Additional file 4H). However, we were unable to observe any significant expression of c-Kit among the luminal cells isolated from C57BL6/J mice, even when using the same c-kit antibody clone (2B8) that was previously used [15]. The c-kit expression levels in FVB mice in our study were quite low when compared with the other studies; one explanation for this discrepancy may be the use of different tissue dissociation protocols.

Immunostaining of sorted populations reveals that both the Sca1^+^CD49b^- ^and Sca1^+^CD49b^+ ^cells express high levels of luminal differentiation markers such as ER and keratin (Krt)18 compared with the Sca1^-^CD49b^+ ^cells (Figure 1D,E). These results demonstrate that there are two functionally distinct types of ER cells in the mammary gland; the vast majority are relatively mature with little proliferation capacity, but a small population representing ~9% of all luminal cells are ER^+ ^progenitors. The ER^+ ^progenitors (Sca1^+^CD49b^+^) express higher transcript levels of luminal differentiation transcripts such as ER, FoxA1 and Gata3 and lower levels of Krt5 and Krt14 when compared with Sca1^-^CD49b^+ ^cells (Figure 1F). Immunostaining of Sca1^-^CD49b^+ ^(ER^-^) cells demonstrates that these cells express lower levels of Krt18 and low but detectable levels of the basal cell-specific Krt5 (Figure 1D,E). These cells express no to low levels of ER, which is in agreement with a previously published report [17]. This intermediate level of expression for both luminal and basal cell markers suggests that these cells are a progenitor cell intermediate between the basal stem cells and the more differentiated ER^+ ^LPs.

The ER^- ^progenitor subpopulation also has relatively higher levels of milk protein transcripts in the virgin state compared with the ER^+ ^progenitor cells, including α-lactalbumin (Lalba) and milk fat globule-epidermal growth factor 8 (Mfg-e8), an observation consistent with previous reports [17]. These cells also express high levels of Elf5 and Lmo4, both of which have been involved with specifying alveolar cell fate (Figure 1F) [18,27]. These results suggest that ER^- ^cells probably represent alveolar progenitors and are primed for milk production.

ALDH is an enzyme family previously reported to identify human mammary stem cells [28]. When we examined ALDH among the mammary cell populations, we observed that all of the ER^- ^and a subset of the ER^+ ^LPs show high levels of enzyme activity, whereas the basal cells (EpCAM^lo^CD49f^hi^) and NCL (Sca1^+^CD49b^-^) ER^+ ^cells show low or absent activity (Additional file 4I).

### Luminal cell population in the mouse mammary gland is relatively deficient in mammary repopulating units

**Figure 2:**
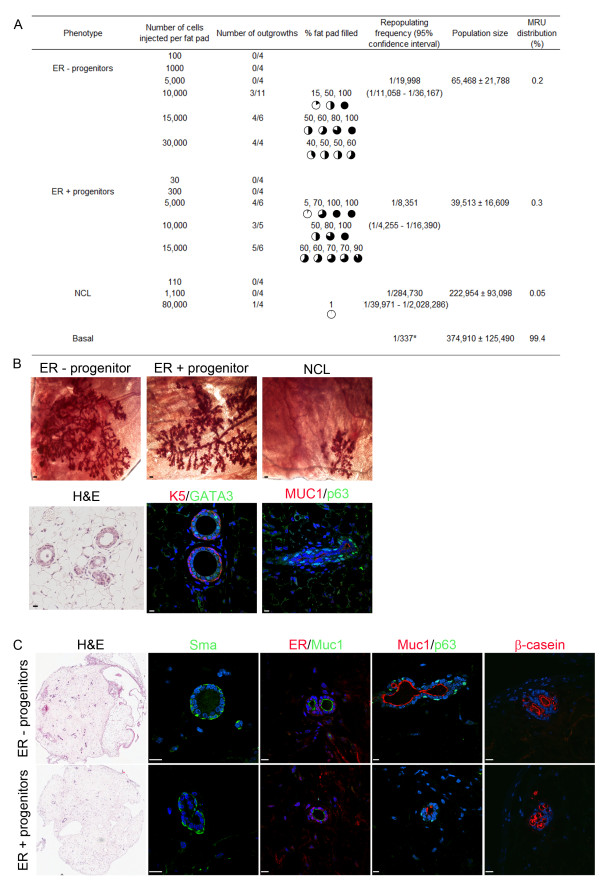
**Mouse luminal progenitors have multilineage differentiation potential but a rare mammary repopulating unit potential**. **(A) **Double-sorted oestrogen receptor-negative (ER^-^) progenitors, oestrogen receptor-positive (ER^+^) progenitors and nonclonogenic luminal (NCL) ER^+ ^cells were injected at the indicated numbers into cleared fat pads and the mammary repopulating unit (MRU) frequency and distribution in each subpopulation was determined. *Basal MRU frequency is from Prater MD, Petit V, Russell IA, Giraddi R, Menon S, Schulte R, Deugnier M-A, Glukhova MA and Stingl J (manuscript submitted). **(B) **Representative whole mounts of outgrowths derived from the different luminal cell populations (upper panels). Representative primary outgrowths of H & E and immunostained sections to detect luminal (GATA3 and MUC1) and basal (K5 and p63) cells (lower panels). Scale bar = 100 μm and 10 μm, respectively. **(C) **Phenotype of colonies generated when ER^- ^and ER^+ ^progenitors are cultured in Matrigel. Scale bar = 10 μm.

Previous studies have demonstrated that mammary stem cells are localised within the basal cell compartment of the mouse mammary epithelium since MRU-enriched populations have a basal cell-signature [8,9]. However, a recent report has challenged the notion that basal cells are the most potent stem cells since a subpopulation of MRUs expressing high levels of the luminal cell differentiation marker CD24 can be also be detected [29]. To investigate this further, we double sorted the three luminal cell subpopulations to ensure purity and minimise contamination from other cell types (Additional file 5) and transplanted them into 25% Matrigel at limiting dilutions into cleared mammary fat pads of recipient mice. As shown in Figure 2A, MRUs are present within the luminal population, albeit at exceedingly low frequencies. No robust MRUs could be detected in the NCL cells, although occasional small ductal-lobular structures could be detected at very low frequencies (Figure 2A,B). Outgrowths derived from these LPs are morphologically normal and contain all of the subpopulations as those derived from basal cells, although a skewing in the distribution of luminal and basal cell populations towards the basal cells is observed when analysed by flow cytometry (Figure 2B; see Additional file 6A). Secondary transplantations reveal that four of the six primary outgrowths contained >5 MRUs, indicating that some of the luminal MRUs are potent and have extensive self-renewal capacity and can generate normal glands (Additional file 6B,C). However, when the distribution of MRUs among all of the mammary cell populations is calculated, we observe that approximately 99% of all MRUs present in a mouse mammary gland are localised within the basal cell population (Figure 2A).

Van Keymeulen and colleagues previously demonstrated that flow-sorted luminal cells can contribute to the luminal epithelium upon transplantation into cleared mammary fat pads, but only when co-transplanted with an approximate equivalent number of basal cells [10]. To determine which luminal cell population has this *in vivo *engrafting potential, double-sorted GFP^+ ^luminal populations (ER^- ^progenitors, ER^+ ^progenitors and NCL cells) were mixed with equal numbers of wild-type total mammary epithelial cells such that the final ratio of GFP^+ ^marked luminal cells to wildtype basal cells was approximately 2:1 (Additional file 7). These cell mixtures were then transplanted into cleared mammary fat pads of NSG mice. Outgrowths containing GFP^+ ^cells could be obtained for all mammary luminal cell populations, although, like the transplants described in Figure 2A, the frequency of this event was rare since only 1 in 30,000 to 340,000 sorted GFP^+ ^luminal cells could engraft (Additional file 7). Unlike the previous report by Van Keymeulen and colleagues, the outgrowths generated in these experiments were not lineage-restricted since the engraftments contained both basal and luminal cells that expressed GFP (Additional file 8). Interestingly, when GFP^+ ^cells engrafted, no engraftment by the co-injected wildtype basal and luminal cells was observed, even when nonlimiting numbers of basal cells were transplanted (Additional file 7). Further work is required to reconcile these two studies.

### Mouse mammary epithelial cells are developmentally plastic

In an attempt to establish parent-progeny relationships between the different populations described in Figure 1A, we sorted the different luminal cell populations and seeded them into *in vitro *and *in vivo *assays. When ER^- ^and ER^+ ^progenitor cells are seeded into three-dimensional Matrigel cultures and maintained for 3 weeks, we observed that the ER^- ^progenitors generated translucent alveolar-like structures that contained eosinophilic material in the lumen, whereas the ER^+ ^progenitors generated alveolar-like outgrowths that were optically dense without any deposits (Additional file 6D). These results demonstrate that these two types of progenitors are functionally distinct. However, when these colonies were examined for expression of lineage markers such as Muc1 and p63, we observed that both progenitors can generate colonies that contain both luminal and basal cell lineages (Additional file 6D). When double-sorted ER^- ^and ER^+ ^LPs were seeded within collagen/Matrigel gels or in 100% collagen gels and transplanted under the renal capsule of NSG mice, both populations generated outgrowths that contained both luminal (Muc1^+^ER^+^) and myoepithelial (Sma^+^p63^+^) cells in virgin mice and β-casein^+ ^cells in pregnant mice (Figure 2C; see Additional file 9A). These outgrowths in virgin mice also contained MRUs since cells dissociated from these renal grafts could engraft multiple cleared mammary fat pads (Additional file 9B).

As a further check for high-fidelity sorting, donor cells (GFP^- ^or GFP^+^) were mixed with genetically tagged (GFP^+ ^or GFP^-^) cells (Additional file 9C) and the genotype of the resultant outgrowths was checked by immunohistochemistry for GFP (Additional file 9D). The outgrowths generated were positive for both luminal and basal markers and expressed the appropriate genotype (Additional file 9D,E). These results demonstrate that ER^+ ^LPs have the potential to dedifferentiate to MRUs.

### ER^+ ^luminal progenitors are relatively insensitive to loss of oestrogen and progesterone

Both luminal and basal cells, and particularly mammary stem cells, were previously reported to be susceptible to withdrawal of oestrogen and progesterone [30]. To investigate the effects of oestrogen and progesterone withdrawal on the different subtypes of mammary luminal cells, 8-week-old C57BL6/J were ovariectomised and the change in the number of different mammary cell types was determined 3 weeks later.

**Figure 3:**
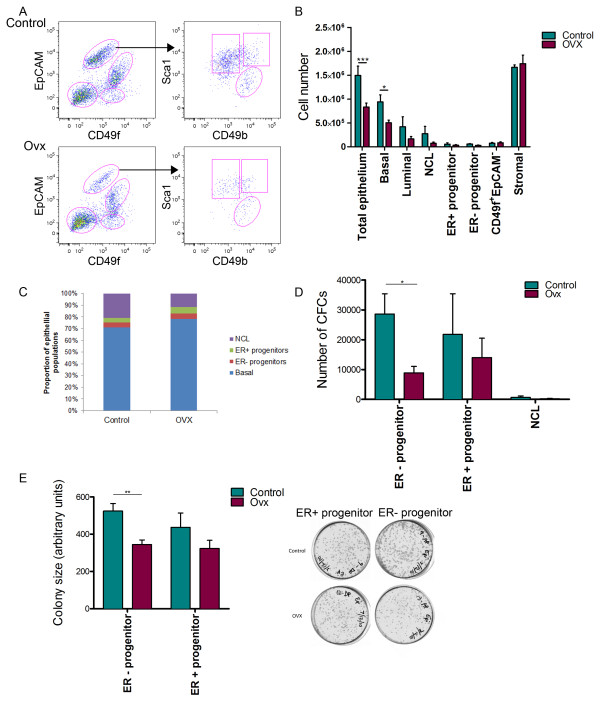
**Influence of ovariectomy on mouse mammary epithelial cell subpopulations**. **(A) **Flow cytometric analysis showing the distribution of epithelial and non-epithelial cell types from control and ovariectomised (Ovx) mice. **(B) **Bar chart depicting total epithelial cell numbers in control and Ovx mice. **(C) **Proportion of epithelial cell populations in control and Ovx mice. **(D) **Total colony-forming cell (CFC) numbers in control and Ovx mice. **(E) **Size of colonies generated from oestrogen receptor-negative (ER^-^) and oestrogen receptor-positive (ER^+^) progenitors isolated from control and Ovx mice. Right panels: CFCs of a representative experiment. Error bars indicate the standard error of the mean for four independent experiments. *P < 0.05, **P < 0.01, ***P < 0.0001. EpCAM, epithelial cell adhesion molecule; NCL, nonclonogenic luminal.

The NCL cells were acutely sensitive to loss of oestrogen since the size of these populations decreased by 72%, with the proportion of these cells to total epithelial cells decreasing in ovariectomised mice when compared with sham-operated mice (Figure 3A,B,C). The basal, ER^- ^progenitor and ER^+ ^progenitor subpopulations were all mildly sensitive to the withdrawal of oestrogen, decreasing by 46%, 47% and 37%, respectively (Figure 3A,B); no statistically significant effect was observed with any of the luminal populations. When the CFC content from ovariectomised and sham-operated mice was analysed, the ER^- ^progenitor population in ovariectomised mice contained 31% fewer progenitors than control mice, and the colonies that were generated were smaller (Figure 3D,E). The number and size of the resultant colonies derived from ER^+ ^progenitors isolated from ovariectomised mice were marginally smaller when compared with controls, although these differences were not statistically significant. No effect of ovariectomy was observed on any of the non-epithelial (EpCAM^-^CD49f^- ^and EpCAM^-^CD49f^+^) cell populations.

These results demonstrate that all populations of mammary epithelial cells are sensitive to loss of steroid hormones, but the ER^+ ^LPs are only mildly affected and have a survival advantage when compared with the other epithelial cell populations.

### Luminal progenitor compartment in human mammary gland is composed of three distinct cell types

**Figure 4:**
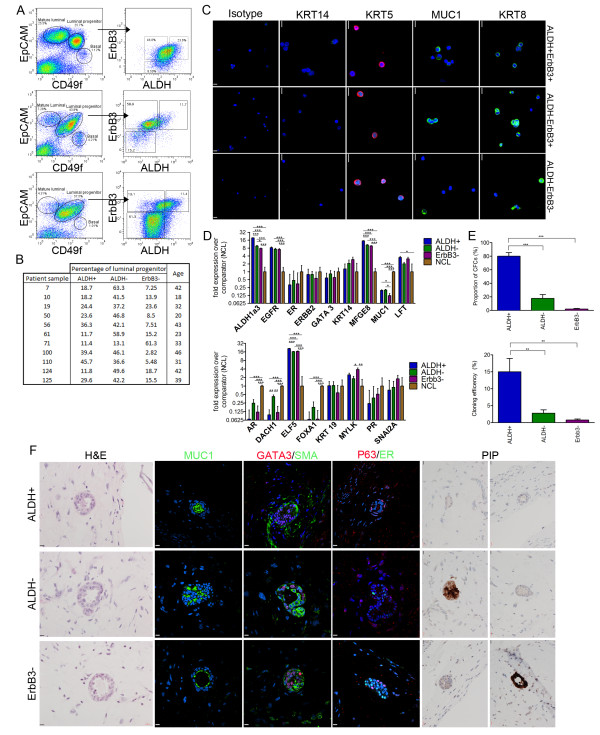
**Three distinct luminal progenitors exist in the human mammary gland**. **(A) **Left: distribution of CD49f and epithelial cell adhesion molecule (EpCAM) in the Lin^- ^population from three patients. Right: distribution of aldehyde dehydrogenase (ALDH) and ERBB3 in the luminal progenitor subset. **(B) **Summary of distribution of luminal progenitor populations and the age of the patients. **(C) **Expression of keratin (KRT)8, MUC1, KRT5 and KRT14 among the three luminal progenitor populations. **(D) **Gene expression analysis of ALDH^+^, ALDH^- ^and ERBB3^- ^subpopulations relative to the comparator populations (nonclonogenic luminal (NCL)) for oestrogen receptor (ER), KRT14, MFG-E8, epidermal growth factor receptor (EGFR), GATA3, ERBB2, MUC1, ALDH1A3, LFT, AR, DACH1, ELF5, FOXA1, KRT19, myosin light chain kinase (MYLK), PR and snail homolog 2a (SNAI2A). **(E) **Frequency and distribution of colony-forming cells (CFCs) among the three luminal progenitor populations. **(F) **H & E and immunostained sections of xenograft gels derived from ALDH^+^, ALDH^- ^and ERBB3^- ^progenitors. Shown is the expression of MUC1, GATA3, SMA, p63, ER and prolactin-induced protein (PIP) among outgrowths. All error bars indicate the standard error of the mean from at least five independent samples. *P < 0.05, **P < 0.01, ***P < 0.0001. Scale bars = 10 μm.

Previous studies have demonstrated that the luminal compartment in the human mammary epithelium can be divided into a luminal-restricted progenitor population (EpCAM^+^CD49f^+^) and mature NCL (EpCAM^+^CD49f **^-^**) cells that express high levels of ER [31,32]. To test the hypothesis that this LP cell compartment is heterogeneous, as in the mouse, we screened the expression of a variety of markers in 11 reduction mammoplasty samples using flow cytometry. We observed that the differential expression of ALDH and ERBB3 was able to resolve the LP population into not two but three subpopulations of cells: ALDH^+^ERBB3^+ ^(ALDH^+^), ALDH^-^ERBB3^+ ^(ALDH^-^) and ALDH^-^ERBB3^- ^(ERBB3^-^) (Figure 4A; see Additional file 2B,C - the threshold between ALDH^- ^and ALDH^+ ^is set with reference to a control population incubated in the presence of ALDH inhibitor DEAB, whilst ERBB3 gating was determined using FMO controls). The proportion of these subtypes of cells is very variable between different donors, especially the ERBB3^- ^subpopulation, which can range in frequency from 2 to 65% of the total LP population (Figure 4B). However, only one-quarter of all patients have a distinctly identifiable ERBB3^- ^population (Figure 4A, middle and lower panels). This population appears to have no correlations with age, although other clinical parameters such as parity, menstrual stage and oral contraceptive use are not known.

All three subpopulations express the luminal-specific KRT8; they also express KRT5 but not KRT14 (Figure 4C). These latter two keratins have historically been considered specific for basal cells, although a recent report has challenged this notion [33]. Gene expression analysis by quantitative RT-PCR confirms that ALDH expression is highest in the ALDH^+ ^population and lowest in the NCL cells (Figure 4D). When we examine the distribution of the luminal differentiation markers MUC1, AR and FOXA1 among the luminal cell populations, we consistently observe that the ERBB3^- ^population displays the lowest levels of luminal differentiation, followed by ALDH^+^, with ALDH^- ^and NCL cells displaying the highest levels of luminal differentiation (Figure 4D) - with the exception being that ALDH^+ ^cells express the highest levels of MUC1 protein (Additional file 10A). Transcripts for ER and GATA3 are not significantly different in the NCL and ALDH^- ^subpopulations when compared with the ERBB3^- ^and ALDH^+ ^subpopulations. The ERBB3^- ^LPs express the highest levels of the basal-specific genes KRT14, myosin light chain kinase and snail homolog 2a and lower levels of the luminal marker KRT19 when compared with the other LP populations. Interestingly, the ALDH^+ ^subpopulation expresses the highest levels of transcripts for ELF5, MFG-E8 and LFT (lactoferrin), thereby suggesting that these cells are primed for milk production (Figure 4D).

It has previously been reported that human breast cancer stem cells often have an EpCAM^+^CD44^+^CD24^-/low ^phenotype [34]. To understand the normal cellular context of this signature, we used flow sorting to determine the distribution of CD24 and CD44 among the different human mammary epithelial cell populations described in Figure 4A. Our results demonstrate that none of the luminal cell populations have this phenotype and that this phenotype is restricted only to the basal cell population (Additional file 10B,C).

To interrogate the proliferative capacities of the three different LP populations, purified cells from each population were seeded into CFC assays. Results demonstrate that the ALDH^+ ^subpopulation had the highest cloning efficiencies and contained the highest proportion of CFCs (Figure 4E). Surprisingly the ERBB3^- ^population had a very low cloning efficiency and contained an almost undetectable number of progenitor cells, which was unexpected since these cells have a relatively undifferentiated phenotype.

These results demonstrate that the ALDH^+ ^subpopulation in the human mammary gland is analogous to the ER^- ^population in the mouse because both populations contain the highest proportion of progenitors and express high levels of ALDH1a3 and alveolar-associated genes (Figures 1C,F and 4D,E; see Additional file 4F).

### Normal human mammary epithelial cells are developmentally plastic

To further characterise the growth and differentiation potential of the three LPs, we sorted these cells and seeded them into collagen gels that were then transplanted under the renal capsule of female NSG mice. All three subpopulations have the ability to generate hollow acinar multilayered structures (Figure 4F), albeit with vastly different efficiencies since the ERBB3^- ^subpopulation generated very few outgrowths. All three LPs gave rise to engraftments that contained both luminal (MUC1^+^GATA3^+^) and basal (p63^+^SMA^+^) cells (Figure 4F). Some engraftments from all populations generated single-layered structures that only contained luminal cells (Additional file 11A). Both ALDH^- ^and ERBB3^- ^progenitors could generate both ER^+ ^and ER^- ^cells (Figure 4F). The ALDH^+ ^cells, despite being able to generate GATA3^+ ^cells, were unable to generate ER^+ ^cells during the initial 5-week assay. However, ER^+ ^progeny could be detected when the assay was extended for an additional 3-week period, thereby suggesting that ALDH^+ ^cells are a primitive progenitor cell that needs additional time to generate all cell lineages (Additional file 11B). A similar pattern of expression was observed when the grafts were examined for expression of prolactin-induced protein, a protein whose expression occurs in the majority of ER^+ ^breast tumours [35]. Both ALDH^- ^and ERBB3^- ^progenitors could generate prolactin-induced protein positive progeny, whereas ALDH^+ ^cells were unable to do so during the 5-week assay (Figure 4F).

### ALDH^+ ^luminal progenitors have a gene signature similar to that obtained from basal-like breast cancers

The LP population has previously been shown to have a gene expression signature resembling that of basal-like breast tumours, while the NCL cells resemble Luminal A/B tumours [32]. We hypothesised that subdividing the LP population would identify a closer relationship between the different types of mammary epithelial cells and the different breast cancer subtypes.

**Figure 5:**
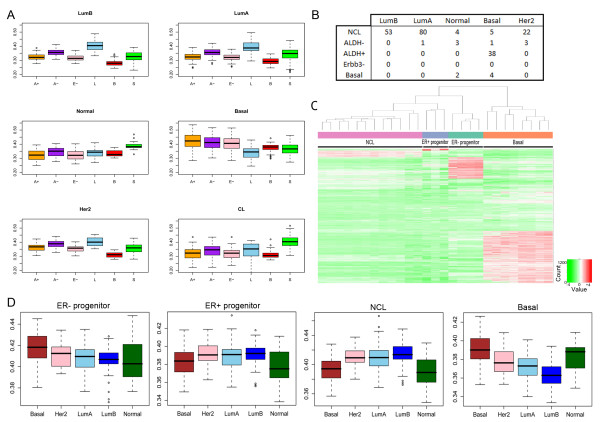
**Comparison of normal mammary cell populations with breast cancer molecular subtypes**. Aldehyde dehydrogenase-positive (ALDH^+^) and oestrogen receptor-negative (ER^-^) progenitors have a gene expression profile similar to basal-like breast cancers. **(A) **Boxplots depicting correlation scores of ALDH^+ ^(A+), ALDH^- ^(A-), ERBB3^- ^(E-), nonclonogenic luminal (NCL; L), basal (B) and stromal (S) cell subtypes, stratified according to breast cancer intrinsic subtype. **(B) **Results of the decision tree nearest-neighbour classifier showing the relation between cell-type-specific signatures and those defining the breast cancer intrinsic subtypes. The ALDH^+ ^subpopulation scores the highest with regards to similarity to the basal-like breast cancer signature. **(C) **Heatmap of genes (red, high expression; green, low expression) of the top 5% differentially expressed genes in the four types of mouse mammary epithelial cells. Samples are clustered on the basis of cell type. **(D) **Boxplots depicting correlation scores of intrinsic subtypes stratified according to mouse mammary cell subtype.

To test this hypothesis we sorted six different freshly isolated mammary cell populations (NCL, ALDH^-^, ALDH^+^, ERBB3^-^, basal and stromal) isolated from up to 11 mammoplasty samples and obtained gene expression profiles of these cells (Additional files 12 and 13). As expected, all three LP populations had gene profiles more similar to basal-like breast cancers than the other sorted breast cell populations (Figure 5A; see Additional file 14). Although the gene signature from the ALDH^- ^population most strongly correlates with basal-like cancers, it also has some correlations with the Luminal A and B signatures (Figure 5A). When we created a decision tree for the different luminal subpopulations, however, we observed that the gene signature of the ALDH^+ ^population, and not the ALDH^- ^or ERBB3^- ^subpopulations, had the highest correlation with those obtained from basal-like breast tumours (Figure 5B). Consistent with what was previously reported, the gene expression signature of the NCL cells resembled those obtained from Luminal A/B breast cancer subtypes and the stromal cells resembled the claudin^low ^subtype (Figure 5A) [32]. To obtain a broader picture of the molecular characteristics of the different mouse mammary epithelial cell subpopulations, gene expression profiles of these cells were obtained. Results showed that the gene signatures of each luminal cell population are unique and distinct from basal cells (Figure 5C; see Additional file 15). Similar observations are seen when the microarray expression profiles of the purified mouse mammary cell populations are compared with those obtained from human breast tumours since the ER^- ^LPs have a gene expression profile that most resembles that of human basal-like breast tumours and the NCL ER^+ ^cells resembling Luminal A/B tumours (Figure 5D).

## Discussion

The results presented here demonstrate that the luminal cell compartment in both the human and mouse mammary glands is much more heterogeneous than initially perceived since progenitors of varying levels of luminal cell differentiation can be identified and prospectively isolated. In the mouse, these populations resolve as separable ER^+ ^and ER^- ^subpopulations, whereas in the human the ALDH^+ ^and ALDH^- ^subpopulations appear to comprise a larger contiguous population. The cell types of the different species appear to be homologous to one another; for example, the ER^- ^LPs in the mouse are equivalent to the ALDH^+ ^cells in the human, and likewise for the ER^+ ^luminal mouse progenitors and the ALDH^- ^luminal human progenitor cells because both populations collectively express higher levels of luminal cell differentiation markers than the ER^-^/ALDH^+ ^subpopulations. The ER^+ ^cells in the mouse are probably ductal-restricted progenitors since they express higher levels of ER and FoxA1, transcription factors that have been demonstrated to be essential for ductal but not lobular morphogenesis during mammary gland development [36]. A similar distribution of FOXA1 is also observed in human ALDH^- ^cells, thereby suggesting that these cells function as ductal progenitors in the human mammary gland. Likewise, the ER^- ^progenitor cells identified in the mouse mammary gland appear to be alveolar progenitors since they express high levels of Elf5 and Lmo4, transcription factors that specify alveolar cell fate [18,27], as well as milk components including Lalba and Mfg-e8 [37]. In the homologous human population, ALDH^+ ^cells express high levels of ELF5 and the milk proteins MFGE8 and LFT, which supports the concept that these cells represent a pool of progenitors that are primed to generate alveoli during pregnancy.

Our results demonstrate that there are two types of ER^+ ^cells in the mammary epithelium; most have little or no proliferative potential and thus are interpreted as being relatively mature, but a small population of ER^+ ^progenitors can be identified and prospectively isolated. Whether these ER^+ ^progenitors give rise to the mature ER^+ ^luminal cells is not known; *in vivo *lineage tracing experiments using an ER^+ ^progenitor-specific promoter will need to be performed to establish the developmental relationships between these two cell types.

Results presented herein demonstrate that ER^+ ^progenitor cells, at least in the normal mouse mammary gland, appear to have a selective advantage over the other mammary epithelial cell populations in adapting to a low-oestrogen environment. ER expression levels in individual mammary epithelial cells are inversely correlated to circulating oestrogen levels [38,39], and thus we hypothesise that the low-oestrogen environment promotes high levels of ER expression and skews the LP pool from an ER^- ^state to an ER^+ ^state. Whether there is a similar preferential survival of ER^+ ^LP cells in the human breast after menopause is not known, although histological studies comparing premenopausal and postmenopausal women report an increase in the frequency of total ER^+ ^cells and proliferating ER^+ ^cells within the postmenopausal mammary gland [40,41]. Garbe and colleagues recently reported that there is an enrichment of LP cells within the mammary epithelium with advancing age. However, these LPs were skewed to a more basal phenotype, which is at odds with our results describing an enrichment of a more luminal type of progenitor [42].

Both oestrogen and progesterone can influence the proliferation of ER^-^/PR^- ^cells in the mammary epithelium via paracrine factors such as amphiregulin, Wnt-4 and RANK ligand [30,43-47]. Progesterone can directly promote the proliferation of PR^+ ^cells via upregulation of cyclin D_1 _and cyclin D_2 _[47]. Evidence also suggests that oestrogen can directly promote the proliferation of ER^+ ^cells in the normal mammary gland since recruitment of ER^+ ^cells into the cell cycle is maximal when circulating oestrogen levels are highest [48]. These ER^+ ^progenitors are of interest because they represent a potential target cell for malignant transformation. ER^+ ^cells are typically distributed within the normal mammary epithelium as single cells, but in atypical ductal hyperplasia and ductal carcinoma *in situ *the ER^+ ^cells tend to be clustered as contiguous islands, suggesting clonal expansion of a mutated ER^+ ^precursor cell. The frequency of proliferating ER^+ ^cells in these islands of cells is positively correlated with breast cancer risk, again suggesting that these ER^+ ^cells are precursors for invasive breast lesions [41].

The hypothesis that postmenopausal breast cancer originates in undifferentiated (ALDH^-^) progenitors does not fit with the fact that these tumours are predominantly of the Luminal A/B subtypes because the ALDH^- ^progenitors described herein have highest correlation with basal-like cancers. One possible explanation for this discrepancy is that tumours may originate in an ALDH^- ^progenitor population, but these cells than differentiate to produce mature ER^+ ^progeny that have a Luminal A/B signature. Another possible explanation is that all of the gene signatures obtained for this study were obtained from premenopausal women (mean age 33.6 years), and that a gene signature of an ALDH^- ^cell in the postmenopausal state could be different (for example, more Luminal A/B-like) than those obtained from premenopausal women. More work in determining the role of these cells in breast cancer progression is clearly required.

ERBB3 is a member of the epidermal growth factor receptor family and often forms heterodimers with ERBB2 [49]. ERBB3 is overexpressed in approximately 22% of breast cancer cases [50], and 25% of cases are reported as being ERBB3-negative. The ERBB3^- ^LPs in the human mammary gland are somewhat unusual since they have an intermediate phenotype between luminal and basal cells, but appear to be deficient in growth potential. Currently unknown is why only one-quarter of patient samples contain this subpopulation and why, when present, the size of this population is so variable. There is no apparent correlation with the age of the tissue donor in the small sample set analysed here. At the time of collection of these tissue samples no information was available regarding parity history, menstrual cycle status and oral contraception use. Further studies with a much larger well-annotated sample set will be required to gain an insight into the nature of these cells.

Although the function of these ERBB3^- ^cells is currently not known, we hypothesise that these cells may be involved with alveologenesis. In the mouse, ErbB3 is required, via phosphoinositide 3-kinase signalling, for the development of the ducts during mammary gland development, but is not essential for the formation of lobules during pregnancy [51,52]. Balko and colleagues reported that ERBB3 expression in the mammary epithelium is highest in the luminal cell populations and lowest in the basal cells, and that loss of ErbB3 in the luminal cell compartment in mice results in an increase in apoptosis of these cells and an expansion of the basal cell population via paracrine signalling [53]. Our results regarding the distribution of ERBB3 among human mammary luminal cells agree with those described by Balko and colleagues, although the presence of a subpopulation of cells with a LP phenotype that are ERBB3^- ^appears to be a novel observation.

We used functional assays to establish the growth and differentiation potential of the different types of human and mouse LP cells, and in agreement with the findings of Keller and colleagues [5] we observed that all types of freshly isolated LPs in both species display multilineage potential when assayed using reconstitution assays, albeit with different frequencies. Other groups have reported that luminal cells in both human and mouse mammary glands can generate multiple lineages of progeny when assayed *in vitro *[17,54-56]. However, these latter observations are not consistent with the results presented here and by Eirew and colleagues [31] since we observe that the majority of cells with *in vivo *engrafting potential are localised within the basal cell compartment. Part of this discrepancy could be due to different groups testing for stemness using different assays. However, the observation that both luminal and basal cell populations exhibit stem cell properties is consistent with a recent report by Van Keymeulen and colleagues. They demonstrate by *in vivo *lineage tracing that both the luminal and basal cell compartments in the mouse mammary gland are maintained by their own stem cell populations during normal tissue homeostasis [10]. This study highlights important caveats in interpreting the results of reconstitution assays, as the differentiation repertoire of cell populations may be perturbed when taken out of a normal tissue environment and purified from other mammary cell types. Reconstitution assays can also mask stem cell potential if cells are transplanted in the absence of appropriate helper cells [10]; this can have obvious limitations when trying to identify putative cancer stem cell populations.

The transplantation process possibly allows LPs to display an expanded differentiation repertoire. Additional work is thus needed to discriminate the growth and differentiation potential that may occur outside normal tissue homeostasis from lineage differentiation that occurs in normal homeostasis. Identifying promoters that are specific for each of the different luminal cell populations will be essential so that the identity of the luminal stem cells and the developmental relationships between the different luminal cells can be established. Such promoters will also be essential for designing transgenic mouse mammary tumour models so that the cell of origin for different molecular types of breast tumours can be established. Current candidates for cell-specific promoters include CD14 for the entire LP population and Elf5 for the ER^- ^LP population. Promoters that are specific for the nonclonogenic ER^+ ^cells and ER^+ ^LPs have yet to be identified.

ALDH has previously been reported to be a marker of breast stem cells [28]. However, we and others have observed that ALDH is expressed primarily in the LP compartment in both humans and mice [57,58]. The expression pattern of ALDH within the LP compartment as opposed to the basal stem cell containing compartment clarifies a discrepancy regarding the influence of the loss of BRCA1 on human epithelial cell differentiation [59]. Women who have lost an allele of BRCA1 have smaller basal cell populations and expanded LP cell populations [32], whereas forced downregulation of BRCA1 results in the expansion of the ALDH^+ ^subpopulation, a population initially interpreted as being the stem cell population and distinct from the LP cells [60]. In hindsight, it is now clear that knockdown of BRCA1 results in expansion of the ALDH^+ ^LP population. In the human epithelium, the ALDH^- ^and ALDH^+ ^subpopulations are clearly part of a larger contiguous population, thereby indicating that these two subpopulations are developmentally tightly linked. We observed that an increase in luminal cell differentiation exists across this population as ALDH expression is lost, and thus one could envision that even low levels of developmental plasticity of an ALDH^- ^cell to an ALDH^+ ^cell could result in loss of luminal cell differentiation.

The results presented here suggest that ALDH1a3 is one of the ALDH isoforms that is being detected by the Aldefluor substrate. Different ALDH isoforms have been shown to be important in different types of cancers, but ALDH1a3 is emerging as a potential cancer stem cell marker in breast cancer [28,61]. ALDH expression, as determined by immunohistochemical staining of tissue sections, has been linked to several breast cancer parameters including ER negativity, high histological grade and general association with basal-like breast cancers [62,63]. Our results using purified subpopulations of human breast epithelial cells are in agreement with these conclusions.

**Figure 6:**
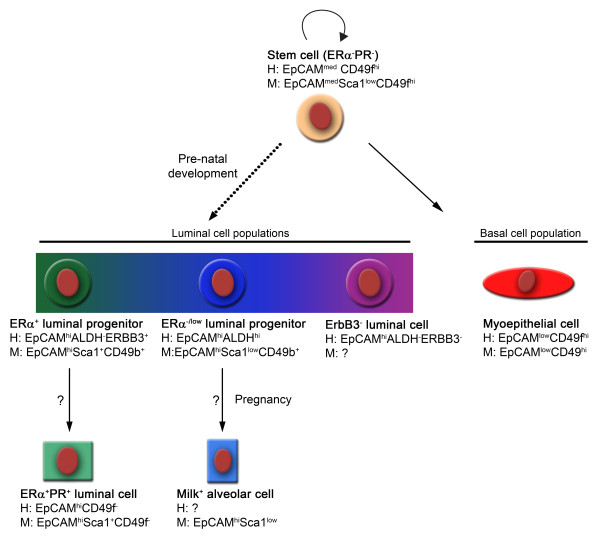
**Proposed epithelial cell hierarchy in the mouse and human mammary glands**. Basal stem cells undergo either self-renewal or differentiation into a luminal progenitor (during embryogenesis) or a myoepithelial cell. Upon certain environmental signals, the luminal progenitor populations may be able to commit to an oestrogen receptor (ER)-positive ductal cell or, during pregnancy, to a milk-producing alveolar cell. *In vivo *lineage tracing experiments in mice will need to be performed to validate these developmental relationships. ALDH, aldehyde dehydrogenase; EpCAM, epithelial cell adhesion molecule; PR, progesterone receptor.

In the context of normal development, therefore, a model of the mammary epithelial cell hierarchy is presented in Figure 6. The basal stem cells undergo either self-renewal or differentiation into a LP (during embryogenesis) or a myoepithelial cell. Environmental signals control commitment of the LP populations to further differentiate to an ER^+ ^ductal cell or, during pregnancy, to a milk-producing alveolar cell. *In vivo *lineage tracing experiments in mice will need to be performed to validate these developmental relationships.

## Conclusion

The results presented in this manuscript demonstrate that there is more heterogeneity present in the luminal mammary epithelium of both humans and mice than initially perceived. LP cells with gene expression patterns consistent with ductal and alveolar progenitors can be identified and prospectively isolated from both species, with these latter cells having gene expression profiles that strongly resemble those obtained from basal-like breast tumours. The LP compartment in the human mammary epithelium also contains an additional cell population that is characterised by lack of ERBB3 expression and low proliferative potential. The ER^+ ^LPs in the mouse are unique in that they are relatively insensitive to loss of oestrogen and progesterone when compared with the other mammary cell populations; this may have implications for the incidence of ER^+ ^and ER^- ^breast cancer in premenopausal women versus postmenopausal women.

## Abbreviations

ALDH: aldehyde dehydrogenase; AF: Alexa Fluor; CFC: colony-forming cell; DMEM: Dulbecco modified Eagle's medium; EpCAM: epithelial cell adhesion molecule; ER: oestrogen receptor; FBS: foetal bovine serum; GFP: green fluorescent protein; H & E: haematoxylin and eosin; Krt: keratin; LP: luminal progenitor; MRU: mammary repopulating unit; NCL: nonclonogenic luminal; NSG: NOD/SCID IL2Rγc^-/-^; PCR: polymerase chain reaction; RT: reverse transcriptase.

## Competing interests

JS is a paid consultant for StemCell Technologies Inc. All remaining authors declare that they have no competing interests.

## Authors' contributions

MS was responsible for conception and design, collection and assembly of data, data analysis and interpretation, and manuscript writing. AT was responsible for data analysis and interpretation. GS, NN, and MP were responsible for collection and assembly of data. IAR was responsible for provision of study material and data interpretation. SA was responsible for conception and design and for data interpretation. PE and CC were responsible for the provision of study material. CJW was responsible for conception and design and for financial support. JS was responsible for conception and design, collection and assembly of data, data analysis and interpretation, and manuscript writing. All authors read and approved the final manuscript.

## Supplementary Material

Additional file 1 **Table S1 presenting antibodies used for immunostaining: primers used for amplification of p53 isoforms and actin by RT-PCR (nested PCRs), and antibodies used to stain the different cell populations**. Table S2 presenting SYBR primers used for quantitative RT-PCR analysis: mouse and human specific primers used for this study. Click here for file

Additional file 2 **Figure presenting the gating cascade**. (A) Gating strategy for flow cytometric analysis and sorting for mouse mammary epithelial cells. Cells were gated on forward (FSC) and side (SSC) scatter to remove debris. Then FSC-W/A and SSC-W/A were selected respectively to obtain single cells. 4',6-diamidino-2-phenylindole (DAPI)-positive and lineage-positive cells were excluded. (B) Gating approach for flow cytometric analysis and sorting for human mammary epithelial cells. Cells were gated on FSC and SSC to remove debris. Then FSC-width and SSC gating were set to exclude doublets. DAPI-positive and lineage-positive cells were excluded. (C) Representative ALDH FACS profiles of total viable cell populations. Cells incubated with ALDH substrate (right) or ALDH and the specific inhibitor DEAB, (left). DEAB controls were used to set the gating strategy to define the ALDH^+ ^population. Click here for file

Additional file 3 **MIAME checklist detailing the microarray experimental information**. Click here for file

Additional file 4 **Figure showing phenotypic characterisation of mouse epithelial cell subpopulations**. (A) Distribution of CD61 among luminal cells. (B) Distribution of CD61 among luminal CFCs showing mean ± standard error of the mean. (C) Proportion of the different luminal subpopulations in virgin and post-involution mammary cells. (D) Effects of antibody staining and flow sorting on colony forming efficiencies. (E) Distribution of CD14 among luminal cells. (F) Distribution of CD14 and Sca1 among luminal (CD24^high^) epithelial cells. (G) Distribution of CD14 and CD24 among luminal CFCs showing mean ± standard error of the mean. (H) Distribution of c-Kit among luminal cells in C57BL6/J (upper panel) and FVB mice (lower panel). (I) Flow cytometric analysis showing the distribution of ALDH among subtypes of mouse mammary epithelial cells. Click here for file

Additional file 5 **Figure showing the gating strategy for double-sorting mouse luminal cell subpopulations**. Click here for file

Additional file 6 **(A) Flow cytometric analysis to determine the distribution of the different epithelial cell populations generated from transplanted ER^+ ^progenitors, ER^- ^progenitors and basal cells**. (B) Number of secondary outgrowth derived from primary transplants of ER^- ^and ER^+ ^progenitors. (C) H & E and immunostained sections of secondary outgrowths. Top panel: H & E section of the entire fat pad and a zoomed image of the black square. Lower panel: sections from outgrowths stained to detect Krt14 and Gata3. (E) Morphology of colonies generated when ER^- ^and ER^+ ^progenitors are cultured in Matrigel. Scale bars = 10 μm. Click here for file

Additional file 7 **Table presenting the MRU frequency and distribution in each GFP^+ ^subpopulation determined for double-sorted ER^- ^progenitors, ER^+ ^progenitors and NCL cells co-injected with wildtype mammary epithelial cells at the indicated numbers into cleared fat pads**. Click here for file

Additional file 8 **Figure showing luminal cells have multilineage potential**. Flow analysis to determine the genotype and distribution of the different epithelial cell populations generated from co-transplanting wildtype mammary cells and (A) GFP^+ ^ER^- ^progenitors, (B) GFP^+ ^ER^+ ^progenitors and (C) GFP^+ ^NCL cells. Far right: GFP^+ ^outgrowths of the initial cell population. Scale bars = 100 μm. Click here for file

Additional file 9 **Figure showing both ER^- ^and ER^+ ^luminal progenitors can have multilineage potential**. (A) Expression of ER, p63, Krt5 and Muc1 among renal graft outgrowths generated in 100% collagen gels. (B) Representative whole mounts of outgrowths generated from ER^- ^and ER^+ ^luminal progenitors initially propagated as subrenal transplants then transplanted into cleared mammary fat pads. Subrenal grafts were dissociated into single cells and all cells injected into the cleared fat pad of secondary recipient mice. Scale bar = 100 μm. Right: bar chart showing the percentage of the fat pad filled by outgrowths 6 to 8 weeks post transplantation (*n *= 4). (C) Schematic illustration of GFP^+/- ^purity check. (D) Representative sections of renal grafts derived from different progenitor types immunostained to detect GFP. (E) Immunofluorescence staining of renal grafts derived from GFP^+ ^donor cells. Sections stained with antibodies to detect Krt5, ER, p63, Muc1, Sma and Gata3. Click here for file

Additional file 10 Figure showing distribution of (A) MUC1, (B) CD24 and (C) CD44 among human mammary epithelial cell subtypes. Click here for file

Additional file 11 **Figure showing immunohistochemistry of xenograft gels derived from ALDH^+^, ALDH^- ^and ERBB3^- ^progenitors for (A) p63 expression and (B) ER expression in engraftments >8 weeks**. Some outgrowths generated from all populations do not contain basal cells. Scale bars = 10 μm. Click here for file

Additional file 12 **Dataset for microarray centroids of purified human mammary cell populations**. Click here for file

Additional file 13 **Dataset for microarray centroids of purified human luminal progenitor cells**. Click here for file

Additional file 14 **Figure showing boxplots depicting correlation scores of ALDH^+ ^(A+), ALDH^- ^(A-), ERBB3^- ^(E-), NCL (L), basal (B) and stromal (S) cell subtypes, stratified according to breast cancer intrinsic subtype from another two cancer datasets: (A) Fridlyand and colleagues **[64]**, and (B) Schmidt and colleagues **[65]. Click here for file

Additional file 15 **Dataset for microarray centroids of purified mouse mammary cell populations**. Click here for file
