# Supplementary material for: Phenotypic and functional characterisation of the luminal cell hierarchy of the mammary gland
Source: Breast Cancer Res. 2012 Oct 22;14(5):R134. doi: 10.1186/bcr3334 (PMC4053112; doi:10.1186/bcr3334)
Supplement: Additional file 1 — Table S1 presenting antibodies used for immunostaining: primers used for amplification of p53 isoforms and actin by RT-PCR (nested PCRs), and antibodies used to stain the different cell populations. Table S2 presenting SYBR primers used for quantitative RT-PCR analysis: mouse and human specific primers used for this study. [file bcr3334-S1.DOCX]

**Table S1. Antibodies used for immunostaining.**

| Primary antibody | Clone | Company | Target species |
| --- | --- | --- | --- |
| CD31-PE/Cy7 | WM-59 | eBioscience | Human |
| CD45-Pe/Cy7 | HI30 | eBioscience | Human |
| EpCAM-PE | 9C4 | Biolegend | Human |
| CD49f-AF647 | GoH3 | Biolegend | Human/Mouse |
| CD49f-Pacific Blue | GoH3 | Biolegend | Human/Mouse |
| Erbb3-Biotin | 1B4C3 | Biolegend | Human |
| CD44-AF647 | IM7 | Biolegend | Human |
| CD24-AF647 | ML5 | Biolegend | Human |
| CD24-PE | M1-69 | eBioscience | Mouse |
| MUC1 | 214D4 | Millipore | Human |
| Muc1 | polyclonal | Abcam | Mouse |
| CD31-Biotin | 390 | eBioscience | Mouse |
| CD45-Biotin | 30-F11 | eBioscience | Mouse |
| Ter119-Biotin | Ter119 | eBioscience | Mouse |
| BP-1-Biotin | 6C3 | eBioscience | Mouse |
| EpCAM-AF647 | G8.8 | Biolegend | Mouse |
| CD49b-PE | HMα2 | Biolegend | Mouse |
| Sca1-PE/Cy7 | D7 | Biolegend | Mouse |
| CD61-AF488 | 2C9.G2 | Biolegend | Mouse |
| CD14-FITC | Sa2-8 | eBioscience | Mouse |
| c-Kit-PE | 2B8 | BD | Mouse |
| ER | 1D5 | Dako | Mouse |
| ER | SP1 | Thermofisher | Human |
| Krt18-Biotin | C-04 | Abcam | Human/Mouse |
| p63 | 4A4 | Abcam | Human/Mouse |
| SMA | polyclonal | Abcam | Human/Mouse |
| Krt14 | polyclonal | Abcam | Human/Mouse |
| Krt5 | polyclonal | Abcam | Human/Mouse |
| Krt8 | M20 | Abcam | Human |
| Gata3 | HG3-31 | Santa Cruz | Human/Mouse |
| PIP (GCDFP15 ) | 23A3 | Novocastra | Human |
| GFP | polyclonal | Abcam | Mouse |
| β-casein | polyclonal | Kind gift from C. Watson | Mouse |

**Table S2. Table of SYBR primers used for qRT-PCR analysis**

| Primer | Forward (5'-3') | Reverse (5'-3') | Target Species |
| --- | --- | --- | --- |
| Aldh1a3 | GCTGACTTGGACTTGGCCGTCG | GCCCCTGCTCCGTTTTGGCA | Mouse |
| CD14 | GCCGCGCGGATTCCTAGTCG | GCGGTGCCGGTTACCTCGAG | Mouse |
| Elf5 | TGCCTTTGAGCATCAGACAG | TACTGGTCGCAGCAGAATTG | Mouse |
| ER | TTACGAAGTGGGCATGATGA | CCTGAAGCACCCATTTCATT | Mouse |
| Foxa1 | GTTGGATGGTTGTGTCGGCCG | CCCTAAGCCCGTGTTGGCGT | Mouse |
| Gata3 | GATGTAAGTCGAGGCCCAAG | GCAGGCATTGCAAAGGTAGT | Mouse |
| Hes1 | ATAGCTCCCGGCATTCCAAG | GCGCGGTATTTCCCCAACA | Mouse |
| Krt14 | TGAGAGCCTCAAGGAGGAGC | TCTCCACATTGACGTCTCCAC | Mouse |
| Krt5 | GAGATCGCCACCTACAGGAA | TCCTCCGTAGCCAGAAGAGA | Mouse |
| Krt8 | AGATCACCACCTACCGCAAG | TGAAGCCAGGGCTAGTGAGT | Mouse |
| Lalba | CAACGGCAGCACAGAGTACGG | TCAGGGCTTCTCACAACGCCA | Mouse |
| Lmo4 | CGGACCGCTTTCTGCTCTATG | CATGCCGCTCTTGGTGTAACA | Mouse |
| Mfg-e8 | GCACCTGCTTGACGGGCCAA | CCCAGCTGTGTAGAACAACCGGTTT | Mouse |
| Ppia | CCTTGGGCCGCGTCTCCTT | CACCCTGGCACATGAATGGTG | Mouse |
| PR | CCAGCATGTCGTCTGAGAAA | GCCTGGCTCTCGTTAGGAA | Mouse |
| Rpl13a | CACTCTGGAGGAGAAACGGAAGG | GCAGGCATGAGGCAAACAGTC | Mouse |
| Stat5a | CGCCAGATGCAAGTGTTGTAT | TCCTGGGGATTATCCAAGTCAAT | Mouse |
| Wnt5a | CAACTGGCAGGACTTTCTCAA | CATCTCCGATGCCGGAACT | Mouse |
| ALDH1A3 | TGTGCGGACGCTGACTTGGAC | GGCATACTCCACGCTCCGCC | Human |
| AR | GCGGGCCAGGAAAGCGACTT | CCCTGGCAGTCTCCAAACGCA | Human |
| DACH1 | TGCCGCATTCTGTCCCTGGT | AGGAAGTTCCAGTCCGACACTTGA | Human |
| EGFR | GCCCCCACTGCGTCAAGACC | ACCTGGCCCAGTGCATCCGT | Human |
| ELF5 | TCTGCCTCACTCCCACAGGGTA | CCACTCCCACACATGGCGCT | Human |
| ER | TGATTGGTCTCGTCTGGCG | CATGCCCTCTACACATTTTCCC | Human |
| ERBB2 | GTCCAGCCGGAGCCATGGGG | TCTGTGCCGGTGCACACTTGG | Human |
| FOXA1 | GAAGATGGAAGGGCATGAAA | GCCTGAGTTCATGTTGCTGA | Human |
| GATA3 | GCCCGGTCCAGCACAGAAGG | AGGGGCCGGTTCTGTCCGTT | Human |
| KRT14 | CCCAGCTCCGCTGCGAGATG | GGGAGGAGGAGAGGTGGGCG | Human |
| KRT19 | ACAGCTGAGCATGAAAGCTGCCT | TCCTGTCCCTCGAGCAGGCT | Human |
| LFT | GCCTCAGGGCTTTTCGGAGCC | AGCCAGACACAGTCCGAGGG | Human |
| MFG-E8 | GGATGCGCCAATCCCCTGGG | GGAGCCCAGGTCCACCTGCA | Human |
| MUC1 | CAGCCAGCGCCTGCCTGAAT | ACCAGAACCCGTAACAACTGTAAGC | Human |
| MYLK | AGGCACCCCCGTGAGGAGAC | ACGGCAAGCCTTTCCACTTGGA | Human |
| PR | CCGCAGGTCTACCCGCCCTA | TGTGCTGCCCTTCCATTGCCC | Human |
| SNAI2A | AGCTGCACTGCGATGCCCAG | GGCTTCTCCCCCGTGTGAGT | Human |
| TBP | GAATATAATCCCAAGCGGTTTG | ACTTCACATCACAGCTCCCC | Human |
| UBC | ATTTGGGTCGCAGTTCTTG | TGCCTTGACATTCTCGATGGT | Human |
