# Supplementary figures and images for: Phenotypic and functional characterisation of the luminal cell hierarchy of the mammary gland
Source: Breast Cancer Res. 2012 Oct 22;14(5):R134. doi: 10.1186/bcr3334 (PMC4053112; doi:10.1186/bcr3334)

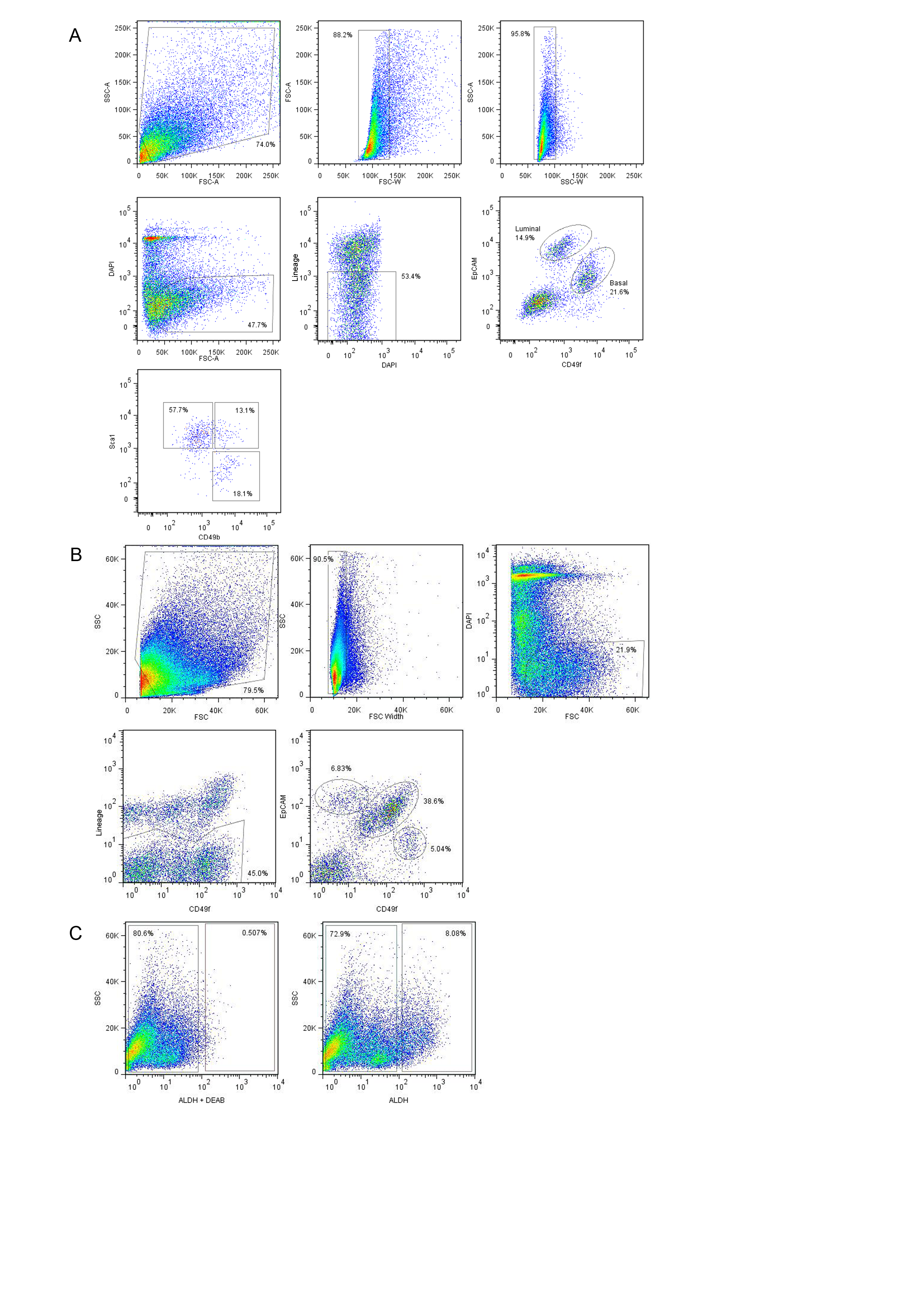

Supplement: Additional file 2 — Figure presenting the gating cascade. (A) Gating strategy for flow cytometric analysis and sorting for mouse mammary epithelial cells. Cells were gated on forward (FSC) and side (SSC) scatter to remove debris. Then FSC-W/A and SSC-W/A were selected respectively to obtain single cells. 4',6-diamidino-2-phenylindole (DAPI)-positive and lineage-positive cells were excluded. (B) Gating approach for flow cytometric analysis and sorting for human mammary epithelial cells. Cells were gated on FSC and SSC to remove debris. Then FSC-width and SSC gating were set to exclude doublets. DAPI-positive and lineage-positive cells were excluded. (C) Representative ALDH FACS profiles of total viable cell populations. Cells incubated with ALDH substrate (right) or ALDH and the specific inhibitor DEAB, (left). DEAB controls were used to set the gating strategy to define the ALDH+ population. [file bcr3334-S2.TIFF]

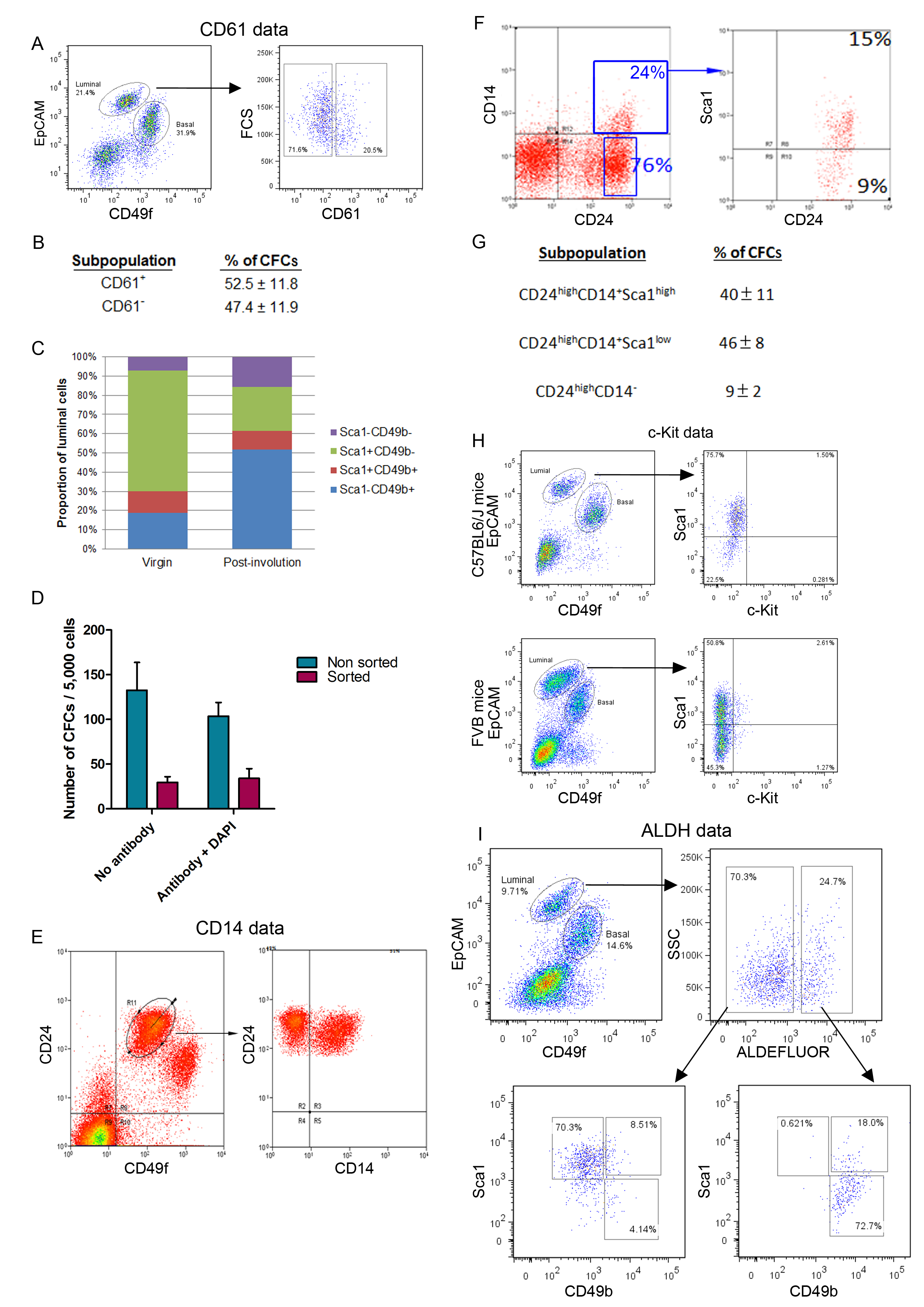

Supplement: Additional file 4 — Figure showing phenotypic characterisation of mouse epithelial cell subpopulations. (A) Distribution of CD61 among luminal cells. (B) Distribution of CD61 among luminal CFCs showing mean ± standard error of the mean. (C) Proportion of the different luminal subpopulations in virgin and post-involution mammary cells. (D) Effects of antibody staining and flow sorting on colony forming efficiencies. (E) Distribution of CD14 among luminal cells. (F) Distribution of CD14 and Sca1 among luminal (CD24high) epithelial cells. (G) Distribution of CD14 and CD24 among luminal CFCs showing mean ± standard error of the mean. (H) Distribution of c-Kit among luminal cells in C57BL6/J (upper panel) and FVB mice (lower panel). (I) Flow cytometric analysis showing the distribution of ALDH among subtypes of mouse mammary epithelial cells. [file bcr3334-S4.TIFF]

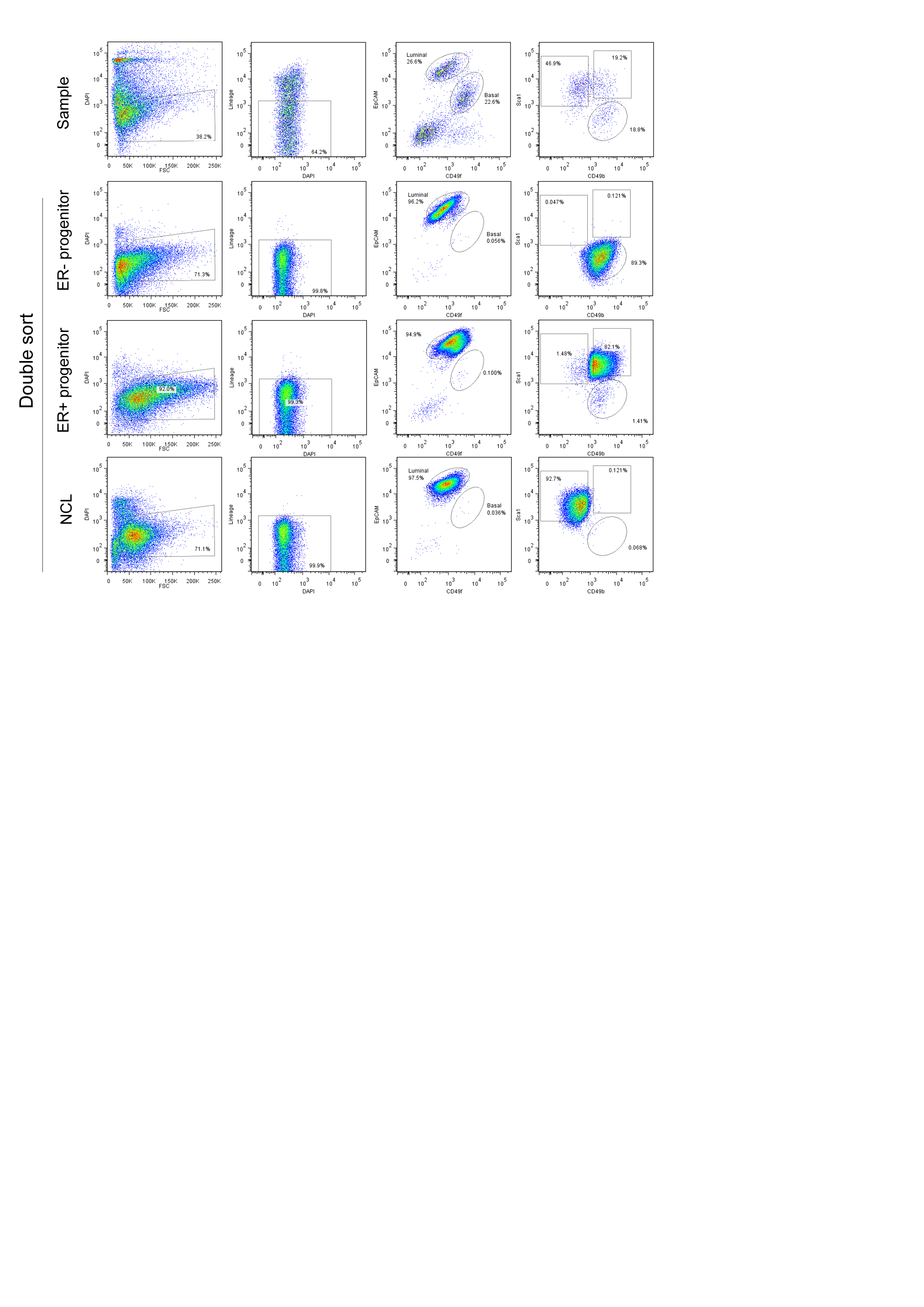

Supplement: Additional file 5 — Figure showing the gating strategy for double-sorting mouse luminal cell subpopulations. [file bcr3334-S5.TIFF]

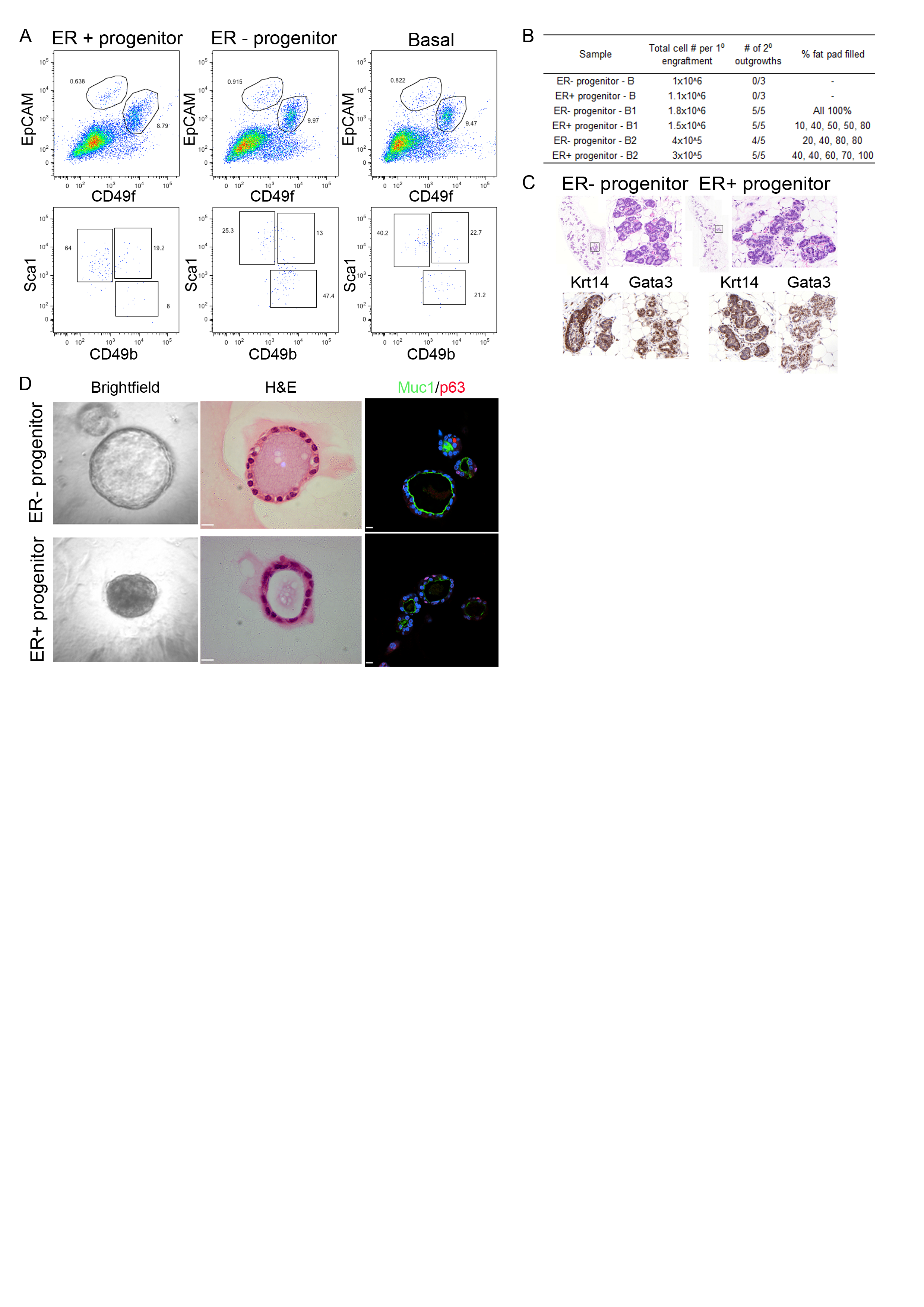

Supplement: Additional file 6 — (A) Flow cytometric analysis to determine the distribution of the different epithelial cell populations generated from transplanted ER+ progenitors, ER- progenitors and basal cells. (B) Number of secondary outgrowth derived from primary transplants of ER- and ER+ progenitors. (C) H & E and immunostained sections of secondary outgrowths. Top panel: H & E section of the entire fat pad and a zoomed image of the black square. Lower panel: sections from outgrowths stained to detect Krt14 and Gata3. (E) Morphology of colonies generated when ER- and ER+ progenitors are cultured in Matrigel. Scale bars = 10 μm. [file bcr3334-S6.TIFF]

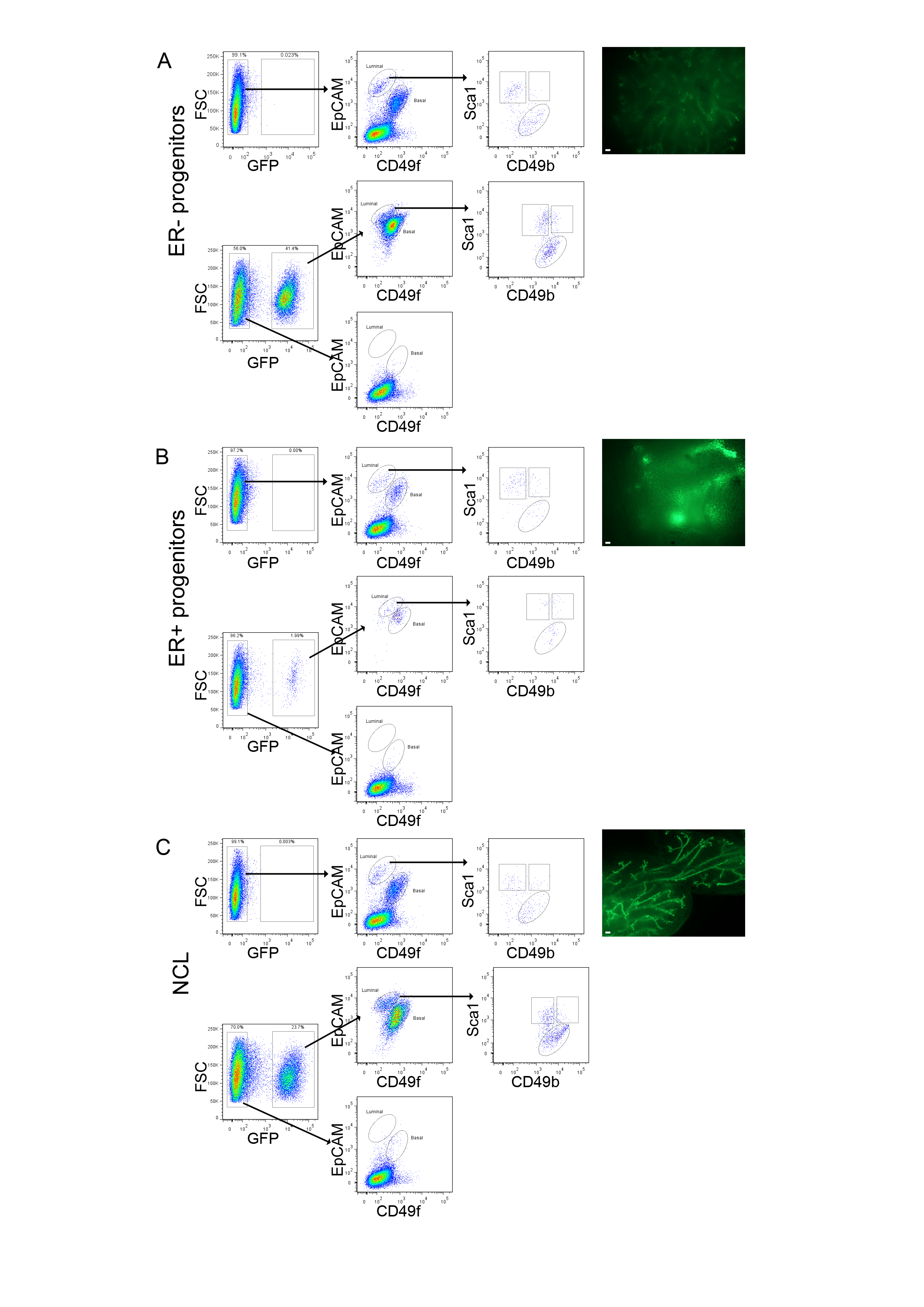

Supplement: Additional file 8 — Figure showing luminal cells have multilineage potential. Flow analysis to determine the genotype and distribution of the different epithelial cell populations generated from co-transplanting wildtype mammary cells and (A) GFP+ ER- progenitors, (B) GFP+ ER+ progenitors and (C) GFP+ NCL cells. Far right: GFP+ outgrowths of the initial cell population. Scale bars = 100 μm. [file bcr3334-S8.TIFF]

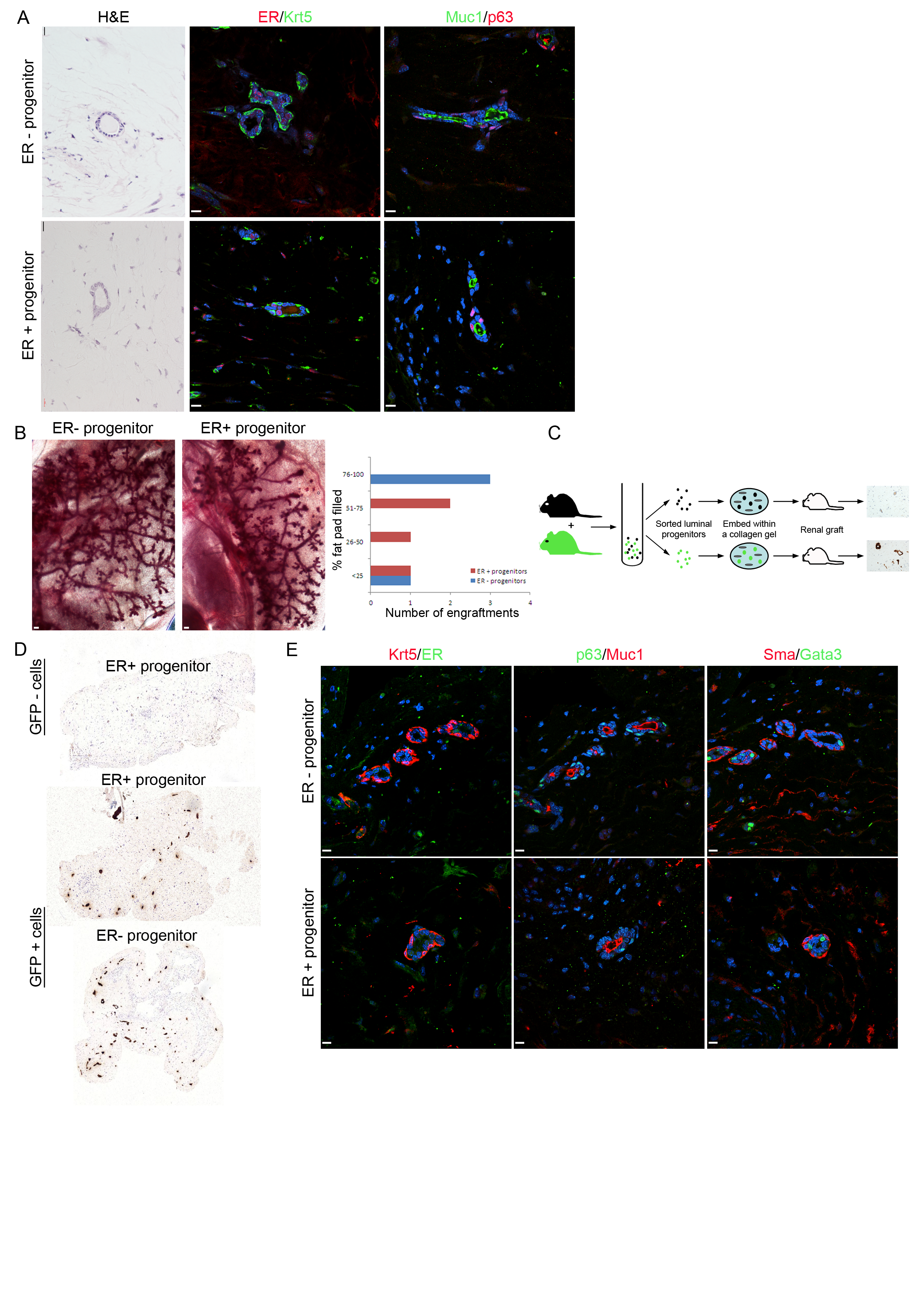

Supplement: Additional file 9 — Figure showing both ER- and ER+ luminal progenitors can have multilineage potential. (A) Expression of ER, p63, Krt5 and Muc1 among renal graft outgrowths generated in 100% collagen gels. (B) Representative whole mounts of outgrowths generated from ER- and ER+ luminal progenitors initially propagated as subrenal transplants then transplanted into cleared mammary fat pads. Subrenal grafts were dissociated into single cells and all cells injected into the cleared fat pad of secondary recipient mice. Scale bar = 100 μm. Right: bar chart showing the percentage of the fat pad filled by outgrowths 6 to 8 weeks post transplantation (n = 4). (C) Schematic illustration of GFP+/- purity check. (D) Representative sections of renal grafts derived from different progenitor types immunostained to detect GFP. (E) Immunofluorescence staining of renal grafts derived from GFP+ donor cells. Sections stained with antibodies to detect Krt5, ER, p63, Muc1, Sma and Gata3. [file bcr3334-S9.TIFF]

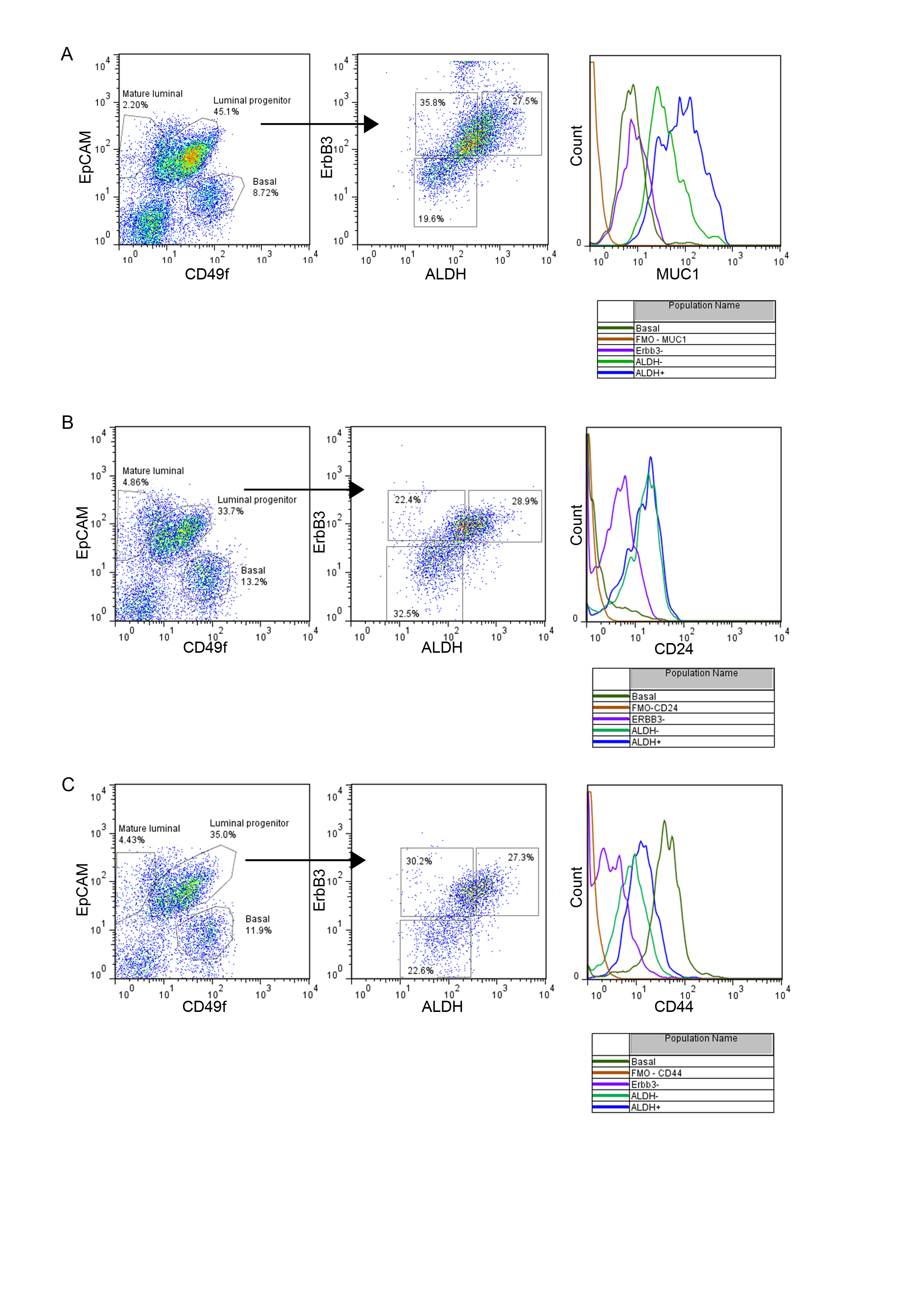

Supplement: Additional file 10 — Figure showing distribution of (A) MUC1, (B) CD24 and (C) CD44 among human mammary epithelial cell subtypes. [file bcr3334-S10.TIFF]

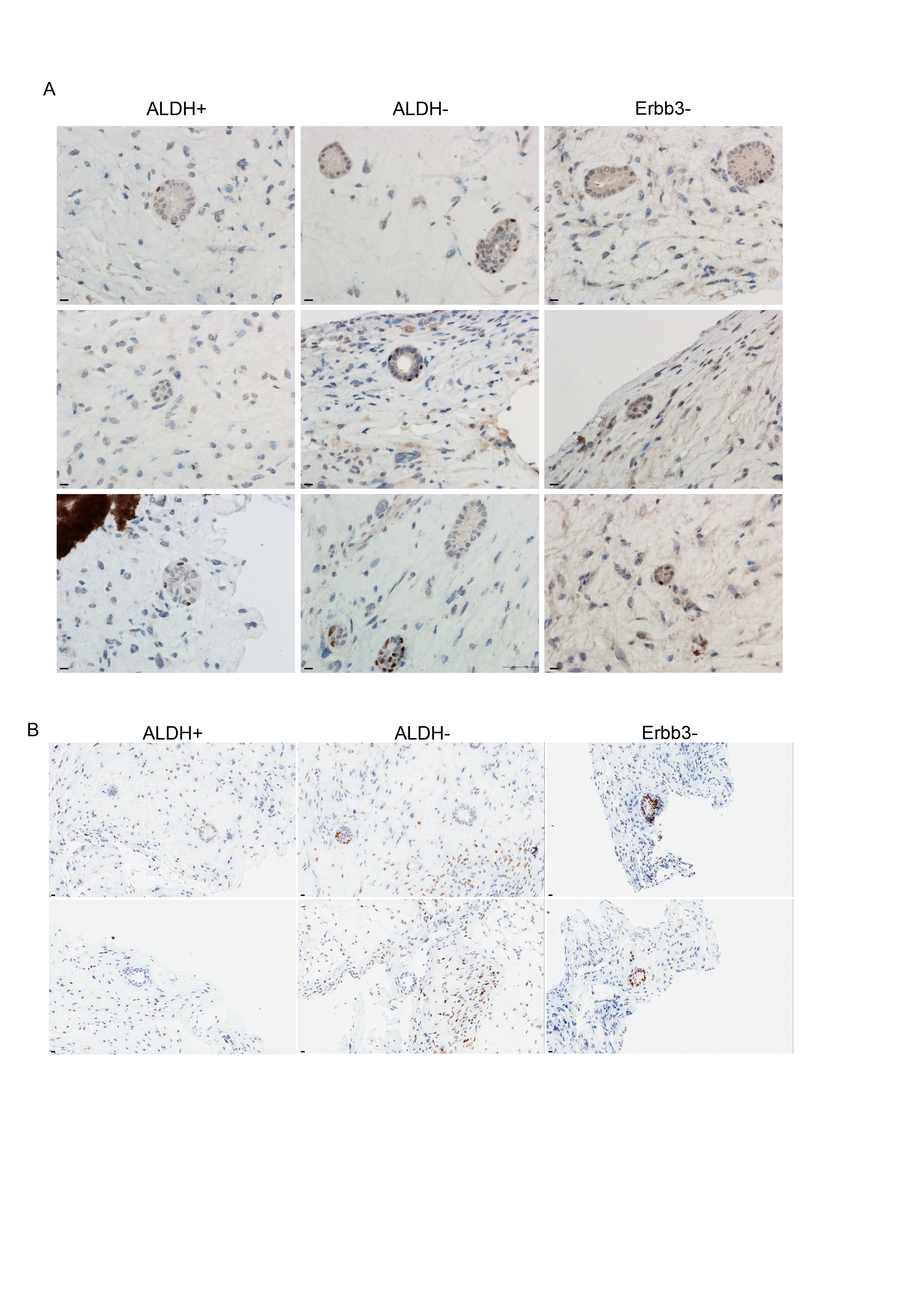

Supplement: Additional file 11 — Figure showing immunohistochemistry of xenograft gels derived from ALDH+, ALDH- and ERBB3- progenitors for (A) p63 expression and (B) ER expression in engraftments >8 weeks. Some outgrowths generated from all populations do not contain basal cells. Scale bars = 10 μm. [file bcr3334-S11.TIFF]

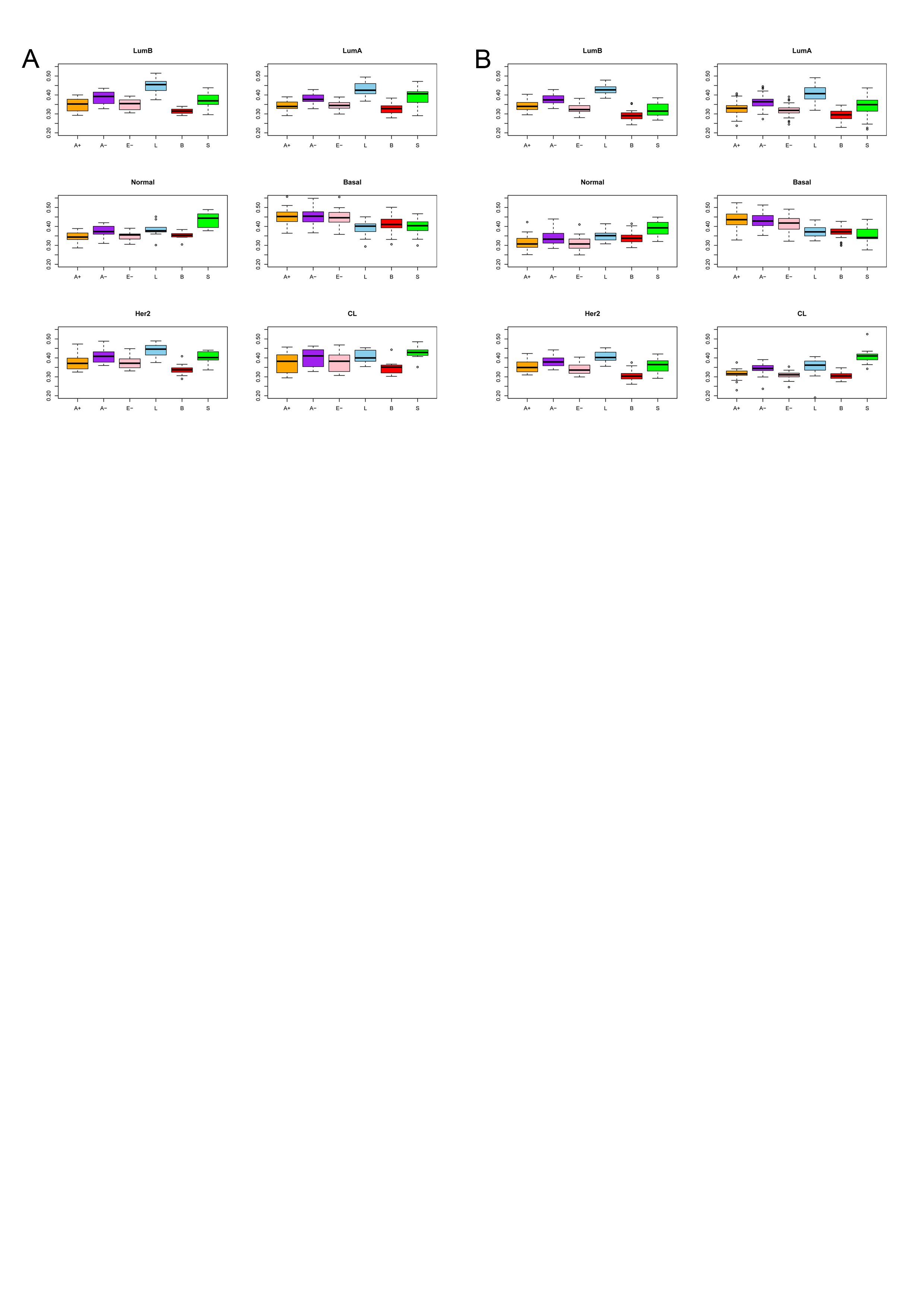

Supplement: Additional file 14 — Figure showing boxplots depicting correlation scores of ALDH+ (A+), ALDH- (A-), ERBB3- (E-), NCL (L), basal (B) and stromal (S) cell subtypes, stratified according to breast cancer intrinsic subtype from another two cancer datasets: (A) Fridlyand and colleagues [64], and (B) Schmidt and colleagues [65]. [file bcr3334-S14.TIFF]
