# Supplementary material for: Phenotypic and functional characterisation of the luminal cell hierarchy of the mammary gland
Source: Breast Cancer Res. 2012 Oct 22;14(5):R134. doi: 10.1186/bcr3334 (PMC4053112; doi:10.1186/bcr3334)
Supplement: Additional file 3 — MIAME checklist detailing the microarray experimental information. [file bcr3334-S3.DOCX]

**MIAME Checklist**

Goal of the experiment: To profile the gene expression of mouse and human mammary luminal cells.

Description of the experiment: Mammary glands from 10-14 week old, female, virgin C57BL/6J mice were dissociated into single cells and luminal subpopulations were sorted by flow cytometry at a purity of ~95%. Human breast tissues from reduction mammoplasties were dissociated into single cells and the luminal subpopulations were flow sorted as described.

Keywords: Cell Type: mouse, human, mammary luminal cells

Experimental design: Mammary glands from 5 C57BL/6J mice were pooled for each experiment. Total RNA was extracted for each group in 4 independent experiments in the mouse and 13 independent patient samples for the human.

Quality control: Total RNA was quality checked using a 2100 Bioanalyser (Agilent) or with cRNA for human samples, where there was not enough sample. All samples had an RNA integrity number ≥ 8.

Public databases: All data files can be accessed via the Gene Expression Omnibus (accession Number: GSE35399).

Origin of each sample: 10-14 week old, female, virgin C57BL/6J mice (Charles River), and discarded tissues from reduction mammoplasties received from Addenbrooke’s hospital.

Manipulation of samples: Human breast tissue or mouse mammary glands were dissociated in collagenase and hyaluronidase for 14-16 h at 37°C, treated with ammonium chloride, trypsin, dispase, DNase and filtered to obtain a single cell suspension. Mammary cells were stained with fluorochrome-conjugated antibodies and the viability dye DAPI and were then passed through a cell sorter.

RNA preparation method for all samples: Total RNA was extracted using a PicoPure RNA extraction kit following the manufacturer’s instructions.

Amplification method: Total RNA was amplified using the Illumina TotalPrep RNA Amplification kit (Ambion).

cRNA hybridisation: Labelled cRNA was hybridised to Illumina BeadArrays overnight following Illumina’s WGGX DirectHyb Assay Guide.

Wash and stain: BeadArrays were washed and stained with streptavidin Cy3 following Illumina’s WGGX DirectHyb Assay Guide.

Illumina scan: BeadArrays were scanned using the AutoLoader and BeadArray scanner at default settings following Illumina’s BeadArray Reader User Guide.

Platform: Illumina Mouse6 v2.0 or the HumanHT12v4 expression BeadChip

Data processing: Data analysis was carried out on R using Bioconductor packages. Raw intensity data from the array scanner were processed using the BASH and HULK algorithms in the beadarray package. Log2 transformation and quantile normalisation of the data was performed. Differential expression analysis was carried out using the limma package.
